# Reprogramming microbial populations using a programmed lysis system to improve chemical production

**DOI:** 10.1038/s41467-021-27226-3

**Published:** 2021-11-25

**Authors:** Wenwen Diao, Liang Guo, Qiang Ding, Cong Gao, Guipeng Hu, Xiulai Chen, Yang Li, Linpei Zhang, Wei Chen, Jian Chen, Liming Liu

**Affiliations:** 1 State Key Laboratory of Food Science and Technology, Jiangnan University, Wuxi, 214122 China; 2 Key Laboratory of Industrial Biotechnology, Ministry of Education, School of Biotechnology, Jiangnan University, Wuxi, 214122 China

**Keywords:** Applied microbiology, Metabolic engineering, Bacterial synthetic biology

## Abstract

Microbial populations are a promising model for achieving microbial cooperation to produce valuable chemicals. However, regulating the phenotypic structure of microbial populations remains challenging. In this study, a programmed lysis system (PLS) is developed to reprogram microbial cooperation to enhance chemical production. First, a colicin M -based lysis unit is constructed to lyse *Escherichia coli*. Then, a programmed switch, based on proteases, is designed to regulate the effective lysis unit time. Next, a PLS is constructed for chemical production by combining the lysis unit with a programmed switch. As a result, poly (lactate-*co*-3-hydroxybutyrate) production is switched from PLH synthesis to PLH release, and the content of free PLH is increased by 283%. Furthermore, butyrate production with *E. coli* consortia is switched from *E. coli* BUT003 to *E. coli* BUT004, thereby increasing butyrate production to 41.61 g/L. These results indicate the applicability of engineered microbial populations for improving the metabolic division of labor to increase the efficiency of microbial cell factories.

## Abstract (web summary)

Microbial ecosystem-based bioproduction requires the regulation of phenotypic structure of microbial populations. Here, the authors report the construction of a programmed lysis system and its ability for reprograming microbial cooperation in poly(lactate-co-3-hydroxybutyrate) and butyrate production by *E. coli* strains.

## Introduction

Synthetic multi-culture populations, which are microbial consortia in a special spatial and temporal environment, maintain a dynamic equilibrium status and can interact with each other to perform desired functions after rational programming^1,2^. Compared to monocultures, the higher robustness and lower burden of multi-culture systems^3,4^ can overcome the limitations inherent in a chassis^5,6^ and achieve metabolic division of labor (DOL) through microbial cooperation^7,8^. Thus, synthetic populations can utilize complex substrates (such as lignocellulose)^9,10^ and complex metabolic pathways (such as biosynthesis of anthocyanins)^11,12^ for bio-production. For example, a lactate platform consisting of *Lactobacillus pentosus*, *Clostridium tyrobutyricum*, *Megasphaera elsdenii*, and *Veillonella criceti* was developed for the direct conversion of lignocellulose to short-chain fatty acids^10^. These results demonstrated the inherent benefits of synthetic populations, such as reducing the metabolic burden from complex reaction cascades and converting complex substrates to valuable chemicals. Although the advantages of spatial cooperation are appealing, temporal cooperation is indispensable to overcoming the limitations of invariant status and further improving the efficiency of multi-culture populations.

Cell lysis provides a direction for achieving temporal cooperation by constructing a structurally changing multi-culture population. In previous research, cell lysis for chemical production mainly focused on the release of macromolecular products to reduce the cost of product purification and simplify downstream processing^13^. For example, an autolysis system based on holin-endolysin and Mg^2+^ was developed to release poly(β-hydroxybutyrate)^14^. Another autolysis system based on a synthetic ribosome binding site was constructed to overcome the difficulty in inducing gene expression at high cell densities^15^. However, besides their application in improving extraction, these autolysis systems have not been used for chemical production. Recently, two programmed populations were constructed using ortholysis circuits^16^ and a stress-gated lysis circuit^17^, and the oscillatory lysis behavior of dominant and dormant strains was observed. This self-lysing strategy provides a possible means for regulating the structure of a population, which is spatially congruent to achieve temporal cooperation.

According to the mechanism of action, lysis systems can be divided into pore-forming^18,19^ and enzymatic^14,20^ systems. Pore-forming lysis systems are based on pore-forming proteins, such as pore-forming toxins^20,21^ which form lesions in biological membranes, and have been used to prevent the establishment of bacterial resistance. However, owing to their complex structures and mechanisms of action, pore-forming lysis systems are less competitive for building synthetic biological tools. Enzymatic lysis systems, such as colicin M (CoIM)^22^, can directly interfere with cell-wall integrity through enzymatic degradation, resulting in cell lysis. The action mode and structural organization of CoIM for lysing *Escherichia coli* are simple and efficient; thus, CoIM has been used in food preservation and for treating infections^23^. However, further studies on how to rationally control CoIM functions to achieve microbial cooperation in microbial populations are still needed.

In this work, microbial cooperation is engineered to improve the efficiency of bio-production by *E. coli*. To this end, a reversibly controllable lysis unit is reconstructed by fusing the C-terminal domain of CoIM with pelB and a protease cleavage site. Then, cellular status-based programmed switches are developed to achieve autonomous decision-making and delay protein activity. Next, a programmed lysis system (PLS) with a lysis unit and programmed switch is reconstructed and used to reprogram microbial populations to achieve DOL. The rationally reprogrammed microbial populations presented here exhibit superior applicability to further increase the efficiency of chemical production.

## Results

### Screening and design of lysis unit

**Fig. 1:**
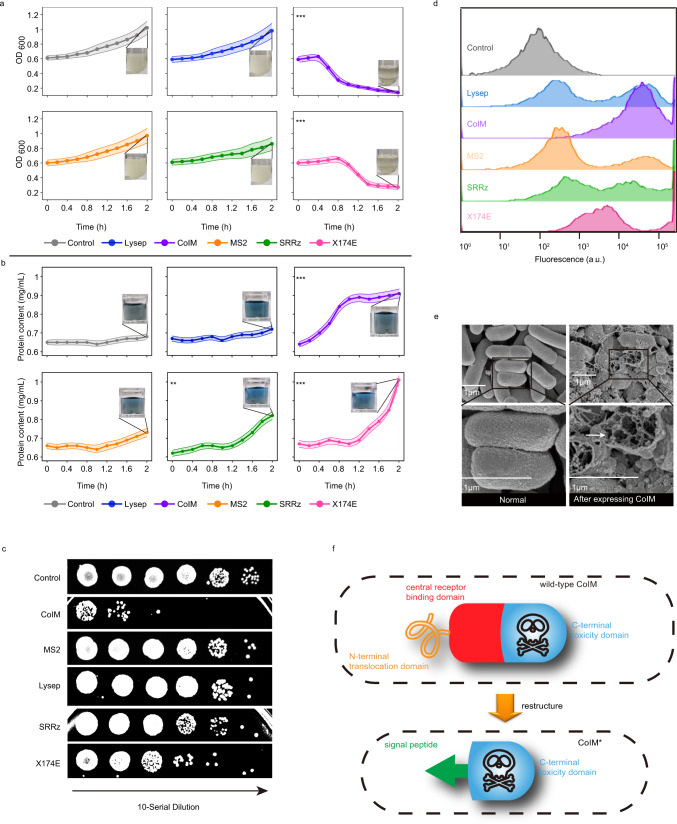
Construction and characterization of lysis unit. **a** The effect of different lysis proteins on cell OD_600_ in 2 h. The clarity of LB medium represented *E. coli* has been lysed by lysis proteins. (*P* values = 0.6938725; 0.000165; 0.6108144; 0.1437364; 0.000358) **b** The effect of different lysis proteins on the release of contents in 2 h. The blue color of the reaction solution represented intracellular protein has been released into the supernatant. (*P* values = 0.0773454; 0.0002937; 0.0582133; 0.0015429; 0.0000676.) Strain JM109 harboring empty vector was used as the control group. All groups were grown at 37 °C and 200 r.p.m. in LB medium for 4 h. Statistical significance of values at 2 h was determined and was indicated as * for *P* < 0.05, ** for *P* < 0.01 and *** for *P* < 0.001, respectively. **c** Spot assays were used to test the effect of different lysis proteins on the viability of *E. coli*. The dilution of samples from left to right was 10^0^, 10^1^, 10^2^, 10^3^, 10^4^, 10^5^. Strain JM109 harboring empty vector was used as the control group. **d** PI staining was used to detect the effect of different lysis proteins on mortality rate. The percentage of fluorescence intensity represented the mortality ratio. The fluorescence intensity of living cells was less than 10^3^, and that of dead cells was more than 10^3^. Strain JM109 harboring empty vector was used as the control group. **e** Scanning electron microscope was used to detect changes on the surface of *E. coli* after expressing different lysis proteins. The white arrows indicated where the cells are broken. Three experiments were repeated independently with similar results. **f** Structure of wild-type CoIM and schematic of reconstructing CoIM*. Values are shown as mean ± s.d. from three (*n* = 3) biological replicates. Two-tailed *t* tests were used to determine statistical significance. Source data are provided as a Source Data file.

To obtain an efficient and quick-response cell lysis system, five lysis proteins, CoIM^24^, Lysep^25^, MS2^26^, SRRz^27^, and X174E^17^, from *E. coli* and phages were selected and overexpressed in *E. coli*. The OD_600_ of cells expressing CoIM decreased from 0.61 to 0.25, which was 192%, 192%, 172%, and 52% lower than the corresponding values in cells expressing Lysep, MS2, SRRz, and X174E, respectively, after 1 h of induction. Similarly, the OD_600_ of cells expressing CoIM was 621%, 629%, 564%, and 93% lower than the corresponding values in cells expressing Lysep, MS2, SRRz, and X174E, respectively, after 2 h of induction. Furthermore, the cell viability decreased from 8.03 × 10^8^ CFU/mL (control group without CoIM expression) to 2.73 × 10^5^ CFU/mL after 2 h of induction (Supplementary Fig. 1). *E. coli* overexpressing CoIM exhibited the least cell growth among the five lysis proteins (Fig. 1c). In addition, it was found that only cells with the CoIM protein could provide a clear broth with no turbidity (Fig. 1a). The protein content of CoIM and X174E in the cell supernatant was 0.87 and 0.94 mg/mL, respectively, after 2 h of induction (Fig. 1b). Accordingly, 93.4% of the cells (control: 5.6%) were damaged, as determined by propidium iodide staining and flow cytometry (Fig. 1d). Cell damage was further demonstrated by scanning electron microscopy, as shown in Fig. 1e. The morphology of cells overexpressing CoIM was irregular or even completely broken. These results demonstrated that CoIM is an efficient and quick-response lysis protein that can release intracellular proteins from *E. coli* via cell lysis and that it is the most suitable element for the lysis unit.

As shown in Fig. 1f, the wild-type CoIM consists of an N-terminal translocation domain, a central receptor-binding domain, and a C-terminal toxicity domain. To increase the lysis efficiency of CoIM, the N-terminal translocation domain, and the central receptor-binding domain were deleted, and then, the C-terminal toxicity domain was fused with different signal peptides and translocated into the periplasmic space to lyse *E. coli*^24^ (Fig. 1f, Supplementary Fig. 2a). Six signal peptides (pelB, NSs, ompA, Tat1, Tat2, and Tat3)^28^ from two secretion pathways (Sec-pathway and Tat-pathway) were introduced and tested. Among them, the C-terminal toxicity domain that fused with pelB to reconstruct CoIM* exhibited the most efficient cell lysis effect, with the OD_600_ decreasing to 0.12 (Supplementary Fig. 2b). The protein content of CoIM* (pelB group) reached 0.92 mg/mL after 2 h, which was only 2% and 3% higher than that of the NSs and ompA groups, respectively (Supplementary Fig. 2c). These results demonstrated that signal peptides from the Sec-pathway are more suitable for reconstructing CoIM, and pelB was selected for further testing because its translocation capacities were 1.86- and 1.15-fold higher than that of the other two signal peptides at 2 h, respectively (Supplementary Fig. 2h).

To efficiently control CoIM*, a protease-trigger mechanism was introduced: the TEVp protease cleavage site (tev site) was fused to the N-terminal of pelB with an F degron (Supplementary Fig. 2d). Following the expression of the protease TEVp, the N-terminal of the F degron was exposed, leading to the degradation and inactivation of the reconstructed CoIM*. As shown in Supplementary Fig. 2e, f, when CoIM* was expressed, the OD_600_ decreased from 0.63 to 0.27, and the protein content in the supernatant increased to 0.93 mg/mL. When CoIM* and TEVp were expressed at the same time, the OD_600_ increased to 1.31, and the protein content was maintained at 0.76 mg/mL. These results indicated that although small amounts of CoIM* were not degraded and partly caused cell lysis due to the high efficiency of the pelB translocation system, the accumulation of TEVp in cells could block CoIM* from entering the periplasmic space and lysing *E. coli*.

### Construction and evaluation of the programmed switch

**Fig. 2:**
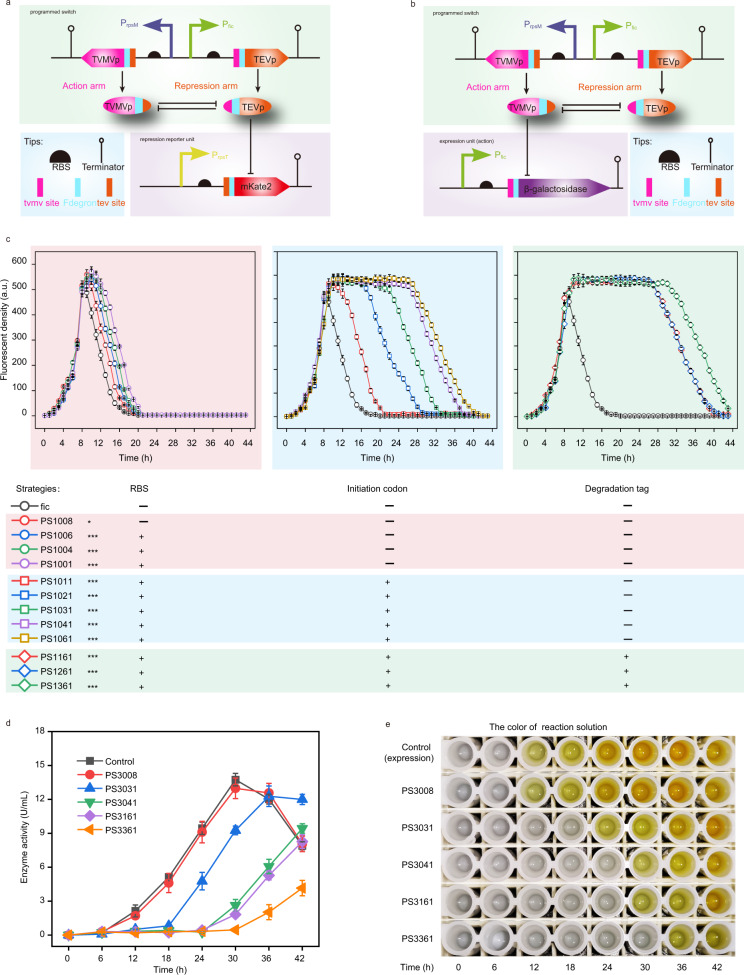
Design, construction, and optimization of the programmed switch. **a** Designing and characterizing the programmed switch. *P*_rpsM_ and *P*_rpsT_ are growth phase promoters; *P*_fic_ is a stationary phase promoter; RBS is a ribosome binding site; TEVp is tobacco etch virus protease; TVMVp is tobacco vein mottling virus protease; mKate2 is a red fluorescent protein. **b** Designing of the programmed switch, and characterizing programmed switch by expression unit. **c** The fluorescence abundance curve of the repression arm was used to characterize the modification effect of different regulation strategies on the programmed switch. (*P* values = 0.016130; 0.000202; 0.000202; 0.002192; 0.002192; 0.000089; 0.000132; 0.000013; 0.000001; 0.000001; 0.000059; 0.000002.) The increase of fluorescence intensity represented the accumulation of mKate2, and the decrease of fluorescence intensity represented the degradation of mKate2 and indicated repression arm began to take effect (programmed switch was activated). After 24 h, 0.1 mL of 100 mg/mL yeast extract was added into medium every 4 h. All groups were grown at 37 °C and 200 r.p.m. in LB medium for 44 h. The OD_600_ and fluorescence intensity were measured every 1 h. Statistical significance of switch was determined and was indicated as * for *P* < 0.05, ** for *P* < 0.01 and *** for *P* < 0.001, respectively. **d**, **e** Programmed switch inhibited enzyme activity of β-galactosidase. The absorbance at 420 nm was measured after cells were sampled each 6 h and lysed by ultrasound. The left **d** was the absorbance curve, and the right **e** was the reaction solution. In strain fic (the control group), TEVp was driven by *P*_fic_. The enzyme activities were measured every 2 h. Values are shown as mean ± s.d. from three (*n* = 3) biological replicates. Two-tailed *t* tests were used to determine statistical significance. Source data are provided as a Source Data file.

*E. coli* expressing CoIM* with stationary phase promoters could not grow (Supplementary Fig. 3). Given their lethal effect on *E. coli*, proteases were used to build a protease-based regulatory switch (programmed switch) to delay the expression of CoIM*. This switch consisted of an action arm and a repression arm. As shown in Fig. 2a, the repression arm expressed the protease TEVp, which is regulated by a more stringent stationary phase promoter (*P*_fic_) (Supplementary Fig. 4c). The action arm expressed the protease TVMVp, which was regulated by a weaker growth phase promoter (*P*_rpsM_) (Fig. 2a, Supplementary Fig. 4f). To construct this programmed switch, these two proteases were modified by fusing their N terminal with an F degron and another protease cleavage site that could be specifically recognized and degraded by the corresponding proteases (Fig. 2a). In the protease-based regulatory switch, protease TEVp could be activated only when TVMVp was completely degraded and vice versa.

First, to characterize the programmed switch behaviors and switch time, a repression reporter unit was constructed, resulting in strain PS1008 (Supplementary Table 8). The repression reporter unit consisted of mKate2, which was modified by fusing its N-terminal with an F degron and a TEVp cleavage site (tev site) that could be specifically recognized and degraded by TEVp (Fig. 2a). The expression of modified mKate2 was under the control of *P*_rpsT_ (a stronger growth phase promoter for producing more mKate2), and modified mKate2 could be degraded by the accumulation of TEVp, but the degradation was removed by cleaving TEVp via TVMVp. As shown in Fig. 2c, the switch time of strain PS1008 was 10 h, indicating that it was delayed by 1 h compared with that of the control group (TEVp was driven by the stationary phase promoter *P*_fic_). These results indicated that TVMVp was rapidly degraded after being cleaved by TEVp. Therefore, three different strategies (RBS regulation, initiation codon regulation, and degradation tag regulation) (Supplementary Fig. 5a–c) were selected to fine-tune TEVp abundance and to regulate the switch time. To verify the effect of these strategies on TEVp abundance, green fluorescent protein (GFP) was fused to TEVp. As shown in Supplementary Fig. 6, the fluorescence intensity gradually decreased, indicating that the abundance of TEVp also decreased. Therefore, as demonstrated in Fig. 2c and Supplementary Fig. 7, when we replaced RBS8 with RBS1 (PS1008–PS1001), the switch time increased from 10 to 11 h. Based on PS1001, when changing the initiation codon from ATG to GGA (PS1011–PS1061), the switch time increased from 11 to 28 h. Based on these two strategies, the degradation tags (DSA, AAV, and LAA) (namely PS1361‒PS1061) were added at the C-terminal of TEVp, causing the switch time to increase to 32 h. In a computational model of the programmed switch (Supplementary Fig. 19), the increase in TEVp abundance (*A*_*t*_) caused the decreased switch time (*T*).

Then, to further characterize the behaviors of the action arm and the relative switch time (when the fluorescence density increased), an action reporter unit was constructed, consisting of mKate2, which was modified by fusing its N-terminal with an F degron and a TVMVp protease cleavage site (tvmv site) that could be specifically recognized and degraded by TVMVp (Supplementary Fig. 8a). The expression of modified mKate2 was under the control of *P*_fic_ (a stationary phase promoter), and modified mKate2 could be degraded by the accumulation of TVMVp, but the degradation was removed by cleaving TVMVp via TEVp. The stationary phase promoter *P*_fic_ was selected to regulate mKate2 expression, resulting in strain PS2008. As shown in Supplementary Fig. 8b, c, when this strain entered the stationary phase, mKate2 fluorescence began to increase after 10 h. Moreover, according to the switch time, four programmed switches were constructed (PS2031, PS2041, PS2161, and PS2361) by replacing the repression reporter unit with the action reporter unit (promoter *P*_rpsT_ was replaced by promoter *P*_fic_, and the tev site was replaced by the tvmv site). The fluorescence intensity began to increase at 20, 27, 28, and 32 h for PS2031, PS2041, PS2161, and PS2361, respectively. These results demonstrated that the programmed switch could also be fine-tuned to obtain a different switch time by fine-tuning the expression of TEVp. Furthermore, to test the universality of the programmed switch, the reporter protein mKate2 was replaced with β-galactosidase, resulting in five strains (PS3008–PS3361). As shown in Fig. 2d, e, both the PS3008 group and the control group began to show enzymatic activity after 12 h (1.7 U/mL), which continued to increase (up to 13.0 U/mL), while the PS3031, PS3041, PS3161, and PS3361 groups did not show enzymatic activity until 24, 30, 30, and 36 h, respectively, suggesting that the results were similar to those of the fluorescence measurements. These results demonstrated that programmed switches were portable and could be used with different genetic components and expression systems. Furthermore, the enzymatic activity of strain PS3361 was the last to appear, indicating that strain PS3361 was the most suitable programmed switch to express CoIM* so that *E. coli* could grow normally at the growth stage.

### Construction and evaluation of a programmed lysis system

**Fig. 3:**
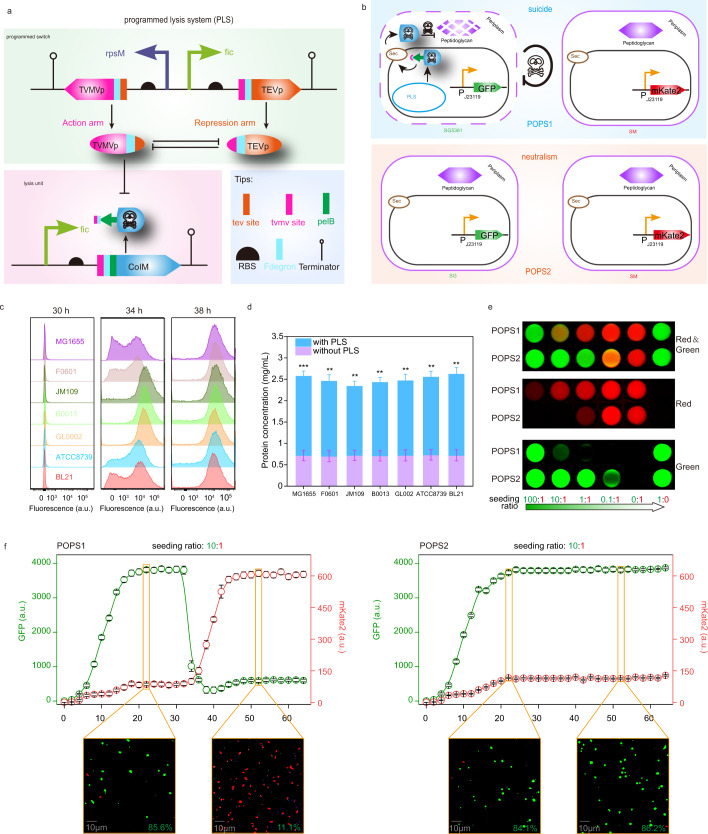
Two applications of programmed lysis system. **a** Schematic diagram of constructing programmed lysis system. **b** Schematic diagram of engineering strains and populations. **c** Mortality ratios comparison of different strains with PLS (30, 34, and 38 h). Mortality ratios of programmed lysis system (PLS) in different *E. coli* were measured by PI staining. The fluorescence intensity of living cells was less than 10^3^, and that of dead cells was more than 10^3^. For each sample, at least 20,000 counts were recorded using a 0.5 mL/s flow rate. All data were exported in FCS3 format and processed using Flow Jo software (FlowJo-V10). **d** Protein content comparison of different strains with/without PLS system. (*P* values = 0.000608; 0.001569; 0.001427; 0.001045; 0.001948; 0.001042; 0.001351.) Statistical significance was indicated as * *P* < 0.05, ** for *P* < 0.01 and *** for *P* < 0.001, respectively. **e** The regulation of seeding ratios and programmed lysis system on population. The green fluorescence intensity represented the accumulation of GFP in strain SG and SG5361. The red fluorescence intensity represented the accumulation of mKate2 in SM. **f** Fluorescence curve of POPS1 and POPS2. It was measured every 2 h and the seeding ratio was 10:1. After 24 h, 0.1 mL of 100 mg/mL yeast extract was added into medium every 4 h. Samples were taken at 22 and 52 h, respectively. Images with green and red fluorescence were also taken by a fluorescence microscope and their colony-forming units (CFUs) were counted to calculate their ratios in the population (the green percentage represented the percentage of green *E. coli*). Values are shown as mean ± s.d. from three (*n* = 3) biological replicates. Two-tailed *t* tests were used to determine statistical significance. Source data are provided as a Source Data file.

A PLS was constructed by integrating a lysis unit (consisting of the reconstructed CoIM*) with a programmed switch (based on PS3361) (Fig. 3a). To demonstrate that the PLS could be effective in different strains of *E. coli*, it was introduced into seven different *E. coli* strains and the results are illustrated in Fig. 3c and Supplementary Fig. 9. The mortality ratios of the *E. coli* strains were <10.0% in the first 30 h (determined by propidium iodide staining), indicating that *E. coli* could grow normally. Four hours later, the mortality ratios increased to a maximum of 94.1% (JM109) and a minimum of 59.0% (BL21) and was over 86.7% at 38 h. Meanwhile, the protein content in the supernatant of all *E. coli* strains was higher than 1.62 mg/mL at 38 h, with a value of only 0.72 mg/mL for the control strain, which did not carry the PLS (Fig. 3d). The mortality ratios of the control groups (strains harboring empty vectors) were maintained at <10.0%. These results indicated that the PLS could efficiently lyse *E. coli* and release cellular inclusions into the broth after 30 h. In addition, the differences in cell growth caused strain-to-strain differences in mortality rate. Firstly, the PLS was based on a stationary phase promoter, which meant that the lysis time of the PLS was related to cell growth. Secondly, the cell growth of strains BL21 and ATCC8739 was slower than that of other strains (Supplementary Fig. 10a). Thus, these two strains were lysed at a slower rate than the other strains. This phenomenon was due to the genotypic differences in different *E. coli* subtypes, causing differences in growth and gene expression.

To determine whether the PLS could regulate the structure of the engineered populations, three different strains, namely SG5361 (carrying the PLS and constitutively expressing GFP), SG (only constitutively expressing GFP), and SM (only constitutively expressing mKate2), were constructed (Fig. 3b). Then, two populations, POPS1 (consisting of SG5361 and SM) and POPS2 (consisting of SG and SM), were reconstructed and cultured for 60 h to detect their structure by fluorescence. When strains SG5361 and SG were seeded at different ratios (100:1, 10:1, 1:1, 0.1:1, 0:1, and 1:0) with respect to strain SM, POPS1, and POPS2 showed different intensities of red and green fluorescence at 60 h (Fig. 3e). When the seeding ratio in populations POPS2 (SG:SM) and POPS1 (SG5361:SM) was 10:1, strain SG accounted for 84.4% of POPS2, whereas strain SG5361 accounted for only 10.6% (Supplementary Fig. 11) after 60 h. As shown in Fig. 3f, when the seeding ratio was 10:1, the red and green fluorescence of POPS2 remained stable after 22 h. However, the green fluorescence decreased from 3804.22 to 481.69, and the red fluorescence increased from 88.59 to 582.48 between 32 and 44 h in POPS1, and then became stable after 44 h. These results indicated that when the seeding ratio was 10:1, the PLS could change the structure of the populations by lysing the original dominant strains, and this structure could remain stable.

### Enhancement of poly(lactate-*co*-3-hydroxybutyrate) release by a PLS

**Fig. 4:**
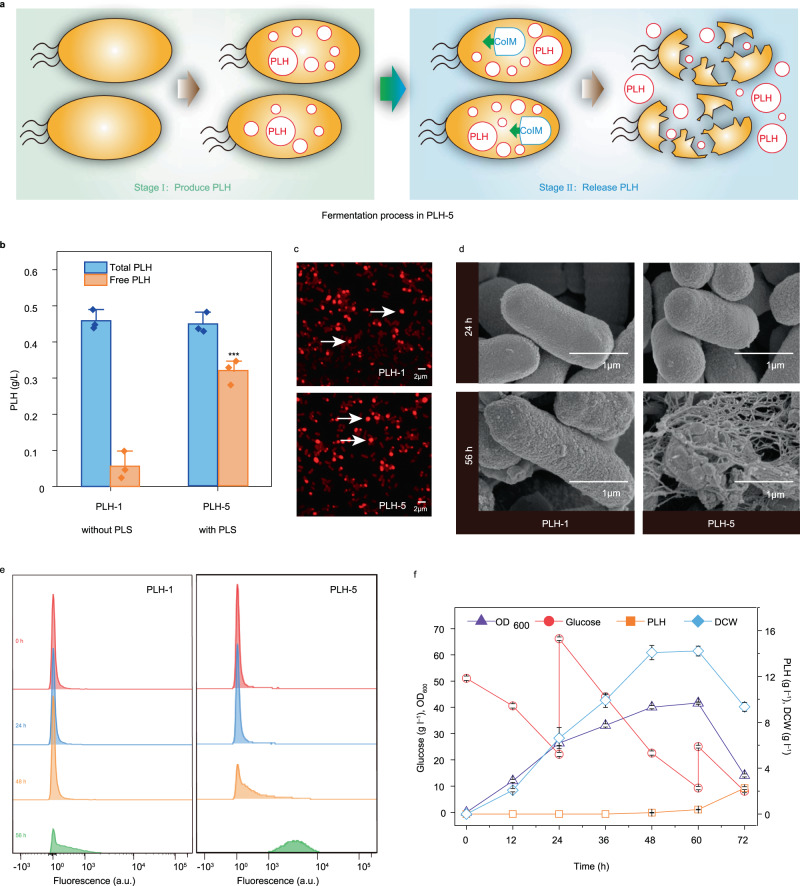
The effect of programmed lysis system on PLH production. **a** The process of producing PLH using programmed lysis system. Stage I was producing PLH and stage II was a programmed lysis system that took effect, lysed *E. coli*, and released PLH. **b** PLH production of engineered populations PLH-1 and PLH-5 in shake flasks. PLH-1 consists of B0032, and PLH-5 consists of B0033 which harbors PLS based on PS3361. PLH released into the medium was defined as ‘free PLH’. (*P* values = 0.655746; 0.000873.) **c** Fluorescence microscopy was used to detect PLH production in cells stained with Nile red. The PLH granule was stained by Nile red. **d** Morphological of PLH-1 and PLH-5. Three experiments were repeated independently with similar results. **e** PI staining to detect cell mortality in fermentation broth at different times. The percentage of fluorescence intensity represented the mortality ratio. The fluorescence intensity of living cells was less than 10^3^, and that of dead cells was more than 10^3^. The fluorescence intensity of living cells was less than 10^3^, and that of dead cells was more than 10^3^. For each sample, at least 20,000 counts were recorded using a 0.5 mL/s flow rate. All data were exported in FCS3 format and processed using Flow Jo software (FlowJo-V10). **f** pH-stat fed-batch cultures of PLH-5 in a 5-L bioreactor. Values are shown as mean ± s.d. from three biological replicates. Values are shown as mean ± s.d. from three (*n* = 3) biological replicates. Two-tailed *t*-tests were used to determine statistical significance. Statistical significance was indicated as * *P* < 0.05, ** for *P* < 0.01 and *** for *P* < 0.001, respectively. Source data are provided as a Source Data file.

Poly(lactate-*co*-3-hydroxybutyrate) (PLH) is a macromolecular polymer that cannot be transported outside the cell after intracellular synthesis^29,30^. Therefore, an ideal solution would enable the release of PLH into the fermentation broth through cell lysis after fermentation is complete. The PLH production process could be divided into two stages (Fig. 4a): (1) PLH synthesis stage in which the precursor’s lactate and acetyl coenzyme A are produced using the original pathway, and four key enzymes, propionyl-CoA transferase (encoded by *pct*), β-ketothiolase (encoded by *phaA*), acetoacetyl-CoA reductase (encoded by *phaB*), and polyhydroxyalkanoate synthase (encoded by *phaC*), are overexpressed to produce PLH; (2) PLH release stage in which cells are lysed by the PLS. For this, the PLS was introduced into strain B0032, a PLH-producing strain reconstructed in a previous study^29^, resulting in the engineered strain B0033 and the population PLH-5 consisting of B0033. As shown in Fig. 4b, 0.45 g/L intracellular PLH was produced by PLH-5 in 56 h, and about 71.21% of the total PLH (0.32 g/L) was released into the fermentation broth. These values were −2% and 82% higher than the corresponding values of PLH-1, respectively. To ascertain the applicability of the PLS to bench-top bioreactors, the performance of PLH-5 (with PLS) in a 5 L fermenter was investigated. We found that 2.26 g/L free PLH was released into the supernatant, which was 335% higher than that of PLH-1 (without PLS) (Supplementary Fig. 12e). The OD_600_ of PLH-5 reached 40.02 at 48 h, which was only 4% lower than that of PLH-1 (Fig. 4f and Supplementary Fig. 12d). In addition, the yield and productivity of PLH-5 improved by 3.68- and 3.83-fold, respectively, compared with those of PLH-1. Thus, the PLS was an effective tool for releasing intracellular products. After changing the programmed switch, we found that the earlier the PLS was activated, the less PLH was produced (Supplementary Fig. 12b, c). Thus, the optimization of lysis time was necessary.

The staining of PLH-1 and PLH-5 (48 h) with Nile red revealed some significant intercellular red spots, indicating that PLH was accumulated (Fig. 4c). At 0–48 h, the OD_600_ of PLH-1 and PLH-5 were the same, but after 48 h, the OD_600_ of PLH-5 gradually decreased to 2.82 (Supplementary Fig. 12a), indicating that PLH-5 was lysed by the PLS. This result was confirmed by scanning electron microscopy, as shown in Fig. 4d. At 24 h, the morphology of PLH-1 and PLH-5 remained elliptical and intact, and only a few cells were damaged in PLH-5. However, at 56 h, most of the cells were damaged in PLH-5. Similarly, the mortality ratio of PLH-1 and PLH-5 was 27.6% and 92.8%, respectively, as determined by propidium iodide staining at 56 h (Fig. 4e). These results demonstrated that the stages of PLH-5 were switched from PLH synthesis to PLH release through the introduction of the PLS and that intracellular PLH could be successfully released without affecting the total PLH titer.

### Enhancement of butyrate production by a PLS

**Fig. 5:**
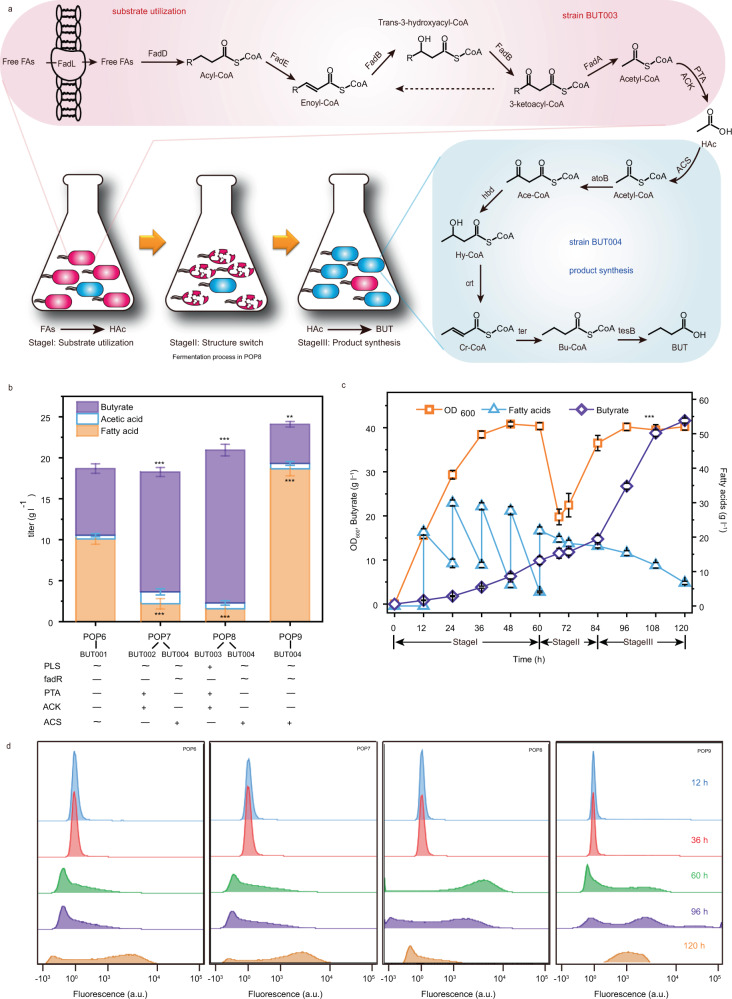
The effect of programmed lysis system on butyrate production. **a** Schematic of the butyrate biosynthetic from fatty acid pathway and process in the fermentation of engineered population POP8. Each gene encodes the following: *FadL*, long-chain fatty acid outer membrane porin; *FadD*, fatty acyl-CoA synthetase; *FadK*, short chain acyl-CoA synthetase; *FadE*, acyl-CoA dehydrogenase; *FadB*, α component of the fatty acid oxidation complex; *FadJ*, α component of the anaerobic fatty acid oxidation complex; *FadA*, β component of the fatty acid oxidation complex; *atoB*, acetoacetyl-CoA thiolase; *hbd*, 3-hydroxybutyryl-CoA dehydrogenase; *crt*, 3-hydroxybutyryl-CoA dehydratase; *ter*, trans-enoyl-CoA reductase; *tesB*, acyl-CoA thioesterase II. **b** The effect of programmed lysis system on producing butyrate. POP6 consists of strain BUT001; POP7 consists of strain BUT002 and strain BUT004; POP8 consists of strain BUT003 and strain BUT004; POP9 consists of strain BUT004. Plus (+) indicates that the corresponding gene or system was introduced. Minus (−) indicates that the corresponding gene was knocked out. Wave (~) indicates that the corresponding gene remained the same. *FadR*, *GntR* family transcriptional regulator, a negative regulator for *fad* regulon and positive regulator of *fabA*; *PTA*, phosphate acetyltransferase; *ACK* phosphate acetyltransferase; *ACS*, medium-chain acyl-CoA synthetase. (*P* values = 0.000425; 0.000168; 0.000057; 0.000185; 0.002112;0.000254.) **c** pH-stat fed-batch culture of POP8 in a 5-L bioreactor. Stages I, II, III were multiple stages during fermentation. (*P* value = 0.000002). **d** PI staining was used to detect mortality ratios in fermentation broth at different times. The percentage of fluorescence intensity represented the mortality ratio. The fluorescence intensity of living cells was less than 10^3^, and that of dead cells was more than 10^3^. For each sample, at least 20,000 counts were recorded using a 0.5 mL/s flow rate. Values are shown as mean ± s.d. from three (*n* = 3) biological replicates. Two-tailed *t* tests were used to determine statistical significance. Statistical significance was indicated as *P < 0.05, ** for *P* < 0.01 and *** for *P* < 0.001, respectively. Source data are provided as a Source Data file.

Using fatty acids as a substrate to synthesize butyrate by engineered *E. coli* causes a huge metabolic burden because the butyrate synthesis pathway contains 11 enzymes^31,32^. An ideal solution to reduce the metabolic burden would be to divide the metabolic pathway into two strains and achieve microbial cooperation. The first strain (strain I) would contain the substrate utilization pathway that utilizes fatty acids to produce acetate, and the second strain (strain II) would contain the product synthesis pathway that utilizes acetate to synthesis butyrate. Therefore, to efficiently produce butyrate from fatty acids, three stages of fermentation were hypothesized (Fig. 5a): Stage I: strain I acts as the dominant strain to utilize fatty acids to produce acetate; Stage II: strain I can be lysed by the PLS, and strain II starts to grow; and Stage III: strain II becomes the new dominant strain to utilize acetate to synthesize butyrate and achieve temporal cooperation with stage I. To validate this hypothesis, four engineered strains were constructed: BUT001, which contained the complete fatty acid degradation (substrate utilization) pathway and butyrate synthesis (product synthesis) pathway; BUT002, which contained only the fatty acid degradation pathway; BUT003 (strain I), which contained the fatty acid degradation pathway and the PLS; and BUT004 (strain II), which contained only the butyrate synthesis pathway (Fig. 5a). Based on these strains, four engineered populations were constructed, namely POP6 (consisting of strain BUT001), POP7 (consisting of strains BUT002 and BUT004), POP8 (consisting of strains BUT003 and BUT004), and POP9 (consisting of strain BUT004).

The fermentation results are illustrated in Fig. 5b. The butyrate titer of POP8 was 18.66 g/L in the shake flask culture, being 56%, 22%, and 74% higher than the corresponding values in POP6 (8.15 g/L), POP7 (14.64 g/L), and POP9 (4.78 g/L), respectively. The residual fatty acid content in POP8 was 1.57 g/L, being 543%, 39%, and 1089% lower than that of POP6 (10.09 g/L), POP7 (2.18 g/L), and POP9 (18.66 g/L), respectively. Moreover, there was only 0.71 g/L acetate in POP8, which was 104% less than that in POP7 (1.45 g/L). These results demonstrated that POP8 could utilize more fatty acids to produce more butyrate, with fewer intermediate metabolites (acetate) remaining. To identify the applicability of the PLS to bench-top bioreactors, the performance of these four populations in 5 L fermenters was investigated. We found that 41.61 g/L butyrates was produced by POP8 (with PLS) (Fig. 5c and Supplementary Fig. 13d–f), which was 115% higher than that of POP6 (without PLS) (19.31 g/L). At 48 h, the OD_600_ of POP8 reached 40.82, which was 0.05-fold higher than that of POP6. In addition, the yield and productivity of POP8 increased by 48% and 115% compared with those of POP6, respectively. Thus, temporal cooperation with the PLS was a suitable strategy for improving butyrate production. When *E. coli* cells were lysed at 12 h, the titer of butyrate was reduced by 66%. This result was attributed to the incomplete fatty acid degradation following premature lysis of BUT003 (Supplementary Fig. 13b, c).

To verify the temporal cooperation that occurred during fermentation, we measured the OD_600_ and cell mortality at different times. As shown in Supplementary Fig. 13a, at 12 h, the OD_600_ of POP6 was only 7.15, being 0.93, 0.97, and 1.00 less than that of POP7 (8.08), POP8 (8.12), and POP9 (8.15), respectively. This indicated that excessive enzyme expression induced a heavier burden and slowed the cell growth rate. After 48 h, the OD_600_ of POP6, POP7, and POP9 remained above 8.18, but that of POP8 decreased at 60 h (6.01) and then recovered to above 8.25, indicating that cell lysis occurred from 48 to 60 h and that the growth resumed after 60 h. As shown in Fig. 5d, <9.8% of the cells were damaged in all populations (POP6, POP7, POP8, and POP9) before 36 h. Twenty-four hours later, cell mortality increased to 80.1% in POP8, but that of POP6, POP7, and POP9 remained at less than 21.1%. This result indicated that the PLS successfully lysed strain BUT003 in POP8. At 96 h, the cell mortality of POP8 decreased to 28.4%, indicating that BUT004 grew and became the dominant strain. In summary, the PLS serves as an advantageous tool for temporal cooperation by changing the phenotypic structure of populations to improve the performance of microbial cell factories.

## Discussion

In this study, a PLS was constructed to regulate the phenotypic structure of microbial populations and to achieve temporal cooperation. To this end, three strategies were implemented: (i) CoIM was reconstructed, and its activity was controlled in a simple and repeatable manner; (ii) a programmed switch was developed, and it achieved an adjustable and delayed response time, and (iii) a PLS was reconstructed to achieve temporal cooperation by reprogramming microbial populations. Upon the implementation of these strategies, PLH and butyrate production showed a large increase of up to 2.26 and 41.61 g/L, respectively, in a 5-L fermenter. These results demonstrated that temporal cooperation using a PLS is a potential strategy for increasing the performance of microbial cell factories.

The CoIM-based lysis unit exhibited superior universality and controllability for lysing *E. coli*. On the one hand, recent studies on the structure and mechanism of action of CoIM revealed that wild-type CoIM consists of three functional domains: (i) the N-terminal translocation domain and (ii) the central FhuA-binding domain can bind to the receptor FhuA and transport CoIM across the outer membrane^33,34^; and (iii) the C-terminal toxicity domain is required for bactericidal activity^22^. Thus, mutations of FhuA and TonB (translocation pathway) can prevent wild-type CoIM from exerting its effects, decreasing the susceptibility of *E. coli* to CoIM. Compared with the wild-type CoIM, the reconstructed CoIM* in the present study (constructed by replacing the N-terminal domain and the central domain with pelB) did not depend on FhuA and TonB, and thus, it had expanded universality. On the other hand, *E. coli* with Cmi (immunity protein of CoIM) could not be lysed because Cmi inhibits the action of CoIM in the periplasm^35^. To achieve *E. coli* lysis, purified CoIM was added as an antimicrobial agent to disrupt cell wall biogenesis^36,37^. Thus, it was necessary to develop another method to achieve automatic lysis by abolishing the inhibitory activity of Cmi. In this study, the activity of the reconstructed CoIM* in the cytoplasm was regulated by a degradable protease^38^. Consequently, tunable lysis in *E. coli* was achieved by controlling the protease input. This strategy extends the universal and controllable regulation of *E. coli* lysis by combining the reconstructed CoIM* with a protease. Based on this, it is possible to regulate the phenotypic structure of microbial populations by CoIM to achieve microbial cooperation. The protease-based programmed switch achieves an adjustable delay response by fine-tuning protease expression, and thus offers an opportunity for smart microbial engineering^39–41^. A programmed switch could automatically and reversibly regulate protein activity. Gene knockout and overexpression are the classic and simplest methods to achieve static regulation and have been used to investigate mechanisms and engineer metabolic pathways^42–45^. However, their irreversibility indicates that they are not suitable for regulating protein activity. In this study, the protease-based programmed switch served as an effective tool to automatically and programmatically regulate protein activity.

Delayed response is a necessary characteristic of a programmed switch. Recently, many genetic^46,47^ and protein^48,49^ circuits have been developed and applied to achieve programmed regulation. However, bio-production is a long-term process, and pathway enzymes are inactive following cell lysis. Thus, it is necessary to delay the response time when regulating the activity of lethal proteins. Previously, in the quorum-sensing system, degradation tags were added to the C-terminal of the target element (post-translational control), and the decrease in GFP was delayed by approximately 5 h^50^. In this study, post-transcriptional and post-translational control were combined to delay the programmed switch time to 33 h. In the programmed switch, growth phase promoters were dependent on sigma-54 to transcript genes during cell growth^51,52^. In addition, stationary phase promoters resulted in the preferential transcription of stationary-phase specific genes by sigma-S and sigma-70^53–55^ in response to environmental signals. Based on these two specific promoters, the product-independent feature of the programmed switch makes it universally applicable to the direct regulation of other proteins.

The PLS provides a strategy to apply cell lysis to production, and achieve metabolic division of labor (DOL)^4^ through temporal cooperation. On the one hand, cell lysis mainly focused on developing autolysis circuits^14^ and self-induced lysis circuits^15^, to simplify downstream processing and save costs. On the other hand, cell lysis based on quorum sensing was used to program the dynamic of microbial consortia. Further, the latter established a paradigm for constructing a variable synthetic microbial consortia^16,17^, which provided a strategy for improving DOL. Recent studies have mainly focused on improving DOL spatially through mathematical models^56,57^, intercellular circuits^58,59^, meta-omics^60,61^, and high-throughput sequencing^62,63^. However, the molecular transport and low biomass of each chassis in microbial populations limit DOL. Thus, temporal cooperation using a PLS is an efficient method to solve these problems. First, to improve the efficiency of spatial cooperation, transporters and efflux systems have been engineered to enhance the transport of intercellular metabolites^64^. In addition, to obtain intracellular products (macromolecular polymers like PLH), additional processes for cell disruption, such as manual lysis (high-pressure homogenization^65^ and the addition of lysozymes^66,67^ and inducers^68,69^), have been performed. Specifically, *E. coli* cells were harvested and then lysed with a typical high-pressure homogenizer by interaction with the fluid and the solid walls of the valve assembly, or enzymes and phages. After cell lysis, the cell debris was removed using a PTFE filter with a pore size of 0.20 mm, and then PLH was extracted with chloroform at 60 °C for 2 days. Next, the flow-through fraction was evaporated, and the precipitant was rinsed with hexane. Finally, PLH was incubated overnight to remove the solvents^70^.

Compared with manual lysis, the application of lysis circuits in a previous study reduced the process of harvesting cells but still required the addition of inducers to induce lysozyme expression. PLH was finally obtained after removing the cell debris, rinsing the precipitant, and incubating. The highest titer, yield, and productivity of PLH in the previous study were 14.4 g/L, 0.36 g/g, and 0.20 g/L/h, respectively^71^. However, the rate of product release with the lysis circuit did not exceed 66.67%^15,68,72^. In our study, the optimized PLS could automatically lyse *E. coli* cells, and the rate of product release with the lysis circuit was up to 71.15%. The final titer, yield, and productivity of PLH were 3.18 g/L, 0.03 g/g, and 0.04 g/L/h, respectively. Thus, the PLS could reduce the costs of equipment for cell lysis, lysozymes and phages for cell lysis, and inducers for gene expression. In addition, the PLS could also reduce time spent on cell harvest, cell lysis, and fermentation in comparison with that of manual lysis.

The limited culture environment ensures that the total biomass of all cells is in dynamic equilibrium. Thus, when the DOL strategy was achieved by spatial cooperation, the biomass of each chassis strain was lower than the total biomass. To increase the biomass of each chassis strain through temporal cooperation, previous studies have mainly focused on optimizing the composition^73^ and environment^74,75^ of multi-culture populations to maximize the performance of the chassis. In a previous study, a recombinant *E. coli* was constructed for butyrate production. The final strain, LW393, produced 33 g/L butyrates in 140 h with glucose as the carbon source^76^. Its yield and productivity were 0.37 g/g and 0.24 g/L/h, respectively. In our study, we applied the PLS to achieve temporal cooperation, and thus the final titer, yield, and productivity of butyrate increased to 41.61 g/L, 0.47 g/g, and 0.35 g/L/h in 120 h, with fatty acids as the carbon source, respectively. The advantages of our research were as follows: on the one hand, fatty acids were degraded into acetate to produce acetyl-CoA for butyrate production, thereby producing fewer by-products. When glucose was used as a carbon source it produces lactate, succinate, and ethanol as by-products^77–79^. On the other hand, temporal cooperation with the PLS reduced the metabolic burden, and thus enhanced the conversion efficiency of each stage.

Taken together, the PLS provides a platform for temporal cooperation, which could simplify the bio-production process and further improve the effectiveness of multi-culture populations. The PLS not only regulates the phenotypic structure of populations but also couples DOL to temporal cooperation. Using this CoIM- and protease-based lysis system, microbial populations could be engineered to improve DOL to boost the performance of microbial cell factories.

## Methods

### Strains and culture conditions

All plasmids used in this study are listed in Supplementary Tables. The DNA manipulations of *E. coli* were performed with Luria–Bertani (LB) medium and plates. The antibiotics were added to a final concentration of 100 mg/L for carbenicillin, 50 mg/L for kanamycin, 50 mg/L for streptomycin, and 50 mg/L for chloramphenicol. IPTG (0.5 mmol/L) or aTc (100 ng/L) was added to induce the expression of enzymes when the cells had grown to an OD_600_ of 0.6–0.8. For multi-culture populations, the seed cultures were diluted to OD_600_ = 3 and mixed proportionally before being transferred. For the PLH production, the seed cultures were grown at 37 °C and 200 r.p.m. in LB medium overnight. Then they were transferred into a 50 mL MR medium containing 50 g L^−1^ glucose and 10 g/L CaCO_3_ as an acid-neutralizing agent. The PLH fed-batch fermentation^80^ was carried out in a 5-L bioreactor with 3 L MR medium. The seed cultures for the fed-batch fermentation were prepared by transferring fresh colony in 50 mL LB medium and culture at 37 °C, 200 r.p.m. and overnight, these seed cultures were inoculated into 100 mL MR medium supplemented with 20 g L^−1^ glucose. And then, after being cultured for 10 h, at 37 °C and 200 r.p.m., the seed cultures were inoculated into a 5 L bioreactor. The pH of the culture was controlled at 7.0 with a mixture of KOH (1.2 mol/L) and KHCO_3_ (2.4 mol/L). For butyrate production, a TB medium was used to grow *E. coli* cells and when cells had grown to an OD_600_ of 0.6–0.8, isopropyl β-d-thiogalactoside (IPTG, 0.4 mmol/L) was added to induce the expression of enzymes. Then the cells were transferred into a 50 ml MR medium containing 20 g/L palmitic acid (emulsified with 2 g/L Brij) and 10 g/L CaCO_3_ as an acid-neutralizing agent. To further improve production, 5 g/L yeast extract was added as an organic nitrogen source. The butyrate fed-batch fermentation was carried out in a 5-L bioreactor containing 3 L MR medium containing 5 g/L yeast extract and 15 g/L glucose. The seed cultures and inoculum concentration were the same as indicated above. After the initial glucose was nearly exhausted, 0.4 mM/L IPTG was added when the OD_600_ reached 10 to induce the expression of enzymes. The carbon source was switched from glucose to FAs at 12 h and suppled 20 g/L every 12 h at stage I. When OD_600_ deceased, 10 g/L glucose was added for cell growth. The pH was controlled at 7.0 with automated adding 1 M NaHCO_3_, and an extra 20 mM NaHCO_3_ was added every 12 h. The stirring rate was set to 600 r.p.m., and the DO content was controlled in the range of 10–20% at stage I and 5–10% at stage III.

### DNA manipulation

Gene deletions were performed with the Red homologous recombination^81^. All plasmids were constructed using the Gibson assembly and basic molecular cloning techniques. The genes *fadD*, *fadE*, *fadB*, and *tesA* were PCR-amplified from the genomic DNA of *E. coli str. K-12 substr. MG1655* for constructing the pathway of FAs utilization. The genes *atoB* and *tesB* were PCR-amplified from the genomic DNA of *E. coli* W3110 for constructing the pathway of butyrate biosynthesis. And genes *crt*, *ter*, and *hbd* were PCR-amplified from the genomic DNA of *Clostridium acetobutylicum*. The genes *ACK*, *PTA*, and *ACS* were PCR-amplified from the genomic DNA of *E. coli str. K-12 substr. MG1655* for the biosynthesis and utilization of acetate. Similarly, the genes *pct* from *M. elsdenii*, *phaA* and *phaB* from *R. eutropha*, and *phaC* from *Pseudomonas sp*. were PCR-amplified for the construction of the PLH biosynthesis pathway. The sequence were provided in Supplementary Table 9.

### Analytical methods

The OD_600_ was determined using a spectrophotometer. Glucose analysis was quantified by the SBA-90E biological sensor^82^. Butyrate and acetate were measured by high-performance liquid chromatography using an Aminex HPX-87H column (7.8 × 300 mm; Bio-Rad) at 45 °C with 5 mM sulfuric acid as the mobile phase. The injection volume was 20 μL and the flow rate was 0.6 mL/min. Samples were collected with centrifugation at 8000 × *g* for 10 min and the supernatant was discarded. And the cells were washed twice with 20 mL distilled water. Dry cell weight (DCW) was assayed after vacuum lyophilization. PLH contents were quantitatively analyzed using gas chromatography (GC-2014, SHIMADZU) after methanolysis of lyophilized cells in chloroform. Analytical P3HB and P(GA-LA) purchased from Sigma-Aldrich were used as analytical standards.

### Protein content

One milliliter sample was taken from LB culture for measuring protein content. Then, they were centrifuged at 5000 × *g* for 5 min and pipetted 150 µL of each sample into the microplate wells. Totally, 150 µL of Pierce Detergent Compatible Bradford Assay Reagent was added to each well, preferably with a multi-channel pipettor, and pipette up and down 4–5 times to mix the sample with reagent. Incubate the plate for 10 min at room temperature and measure the absorbance at 595 nm on a plate reader. At last, subtract the average 595 nm measurement for the blank replicates from the 595 nm measurements of all other individual standards.

### Spot assay

The seed was cultivated in the logarithmic phase and diluted to an absorbance at 660 nm (A660) of 1.0 in PBS. Aliquots (4 μL) of 10-fold serial dilutions were spotted onto LB agar plates. Growth was assessed after incubation for 12 h at 37 °C.

### Light microscopy

One microliter of exponentially growing *E. coli* culture was plated on an LB agar pad in a microscope cavity slide. The fluorescence intensity of GFP was measured using a C-FL-C FITC filter cube (EX, 465–495 nm; DM, 505 nm; BA, 512–558 nm). The fluorescence of mKate2 was measured using a C-FL-C TRITC (tetramethylrhodamine) filter cube (EX, 527–553 nm; DM, 565 nm; BA, 577–633 nm). Microscopy image was obtained using a Nikon ECLIPSE 80i microscope which was equipped with a ×100 oil immersion objective. Brightfield images (exposure, 100 ms), GFP fluorescence images (FITC, exposure, 400–600 ms), and mKate2 fluorescence images (TRITC, exposure, 400–600 ms) were analyzed using Image J software.

### GC analyses

To quantify PLH after cultivation^83^, the sample was frozen in liquid nitrogen for 15 min and lyophilized for 24 h. To quantify the residual P(D-LA-*co*-3HB), ethanolysis of the whole culture was performed, and the concentrations of LA-ethyl and 3HB-ethyl were determined using gas chromatography (GC). Totally, 500 mL of 15% v/v H_2_SO_4_ in ethanol and 250 mL chloroform were added to the dried sample. Then it was heated at 100 °C for 120 min, with a 30 min interval for vortexing. The sample was cooled down to room temperature. Totally, 5 mL of 0 °C ultrapure water was added to the sample, and it was vigorously agitated by vortexing. The sample was centrifuged at 4 °C and 1690 × *g* for 15 min. The lower chloroform layer was separated and dried by passing it through Na_2_SO_4_, and it was incubated with molecular sieves (4A 1/16, Wako) for 30 min. GC-2010 (Shimazu) was coupled with a flame ionization detector which equipped with an InterCap1 column (0.25 mm I.D. × 30 m, d*f* = 0.25 mm; GL Sciences) and a hydrogen generator OPGU-2200 (STEC). The GC program was as follows: N_2_, 43 mL/min; He, 30 mL/min; H_2_, 40 mL/min, air, 400 mL/min; detector temperature, 310 °C; injector temperature, 205 °C; gas temperature, 100 °C; and column oven, 40–300 °C. Poly (50 mol% d,l-lactide-*co*-glycolide) (Mw = 3 × 10^4^–6 × 10^4^; Sigma-Aldrich) and P(3HB) were used as the standard curves for LA and 3HB, respectively.

To quantify the free fatty acids after fermentation, each 200 μL sample was converted to fatty acid methyl esters by using 800 μL 1.8 M H_2_SO_4_ in 90% (V/V) methanol at 70 ± 1 °C for 20 min. After the reaction mixture was cooled, 500 μL hexane was added and followed by vigorous vortexing to extract fatty acid methyl esters. The reaction mixture was centrifuged at 12,000 × *g* to separate the aqueous and the organic layers. At last, the organic layer which contains the products was transferred to GC vials for quantification by using a gas chromatograph (GC; 7890A GC system, Agilent Technologies, Palo Alto, USA) equipped with a flame ionization detector. The GC analysis was carried out with an HP-88 column (Agilent Technologies) with 0.2 μm film thickness, 0.25 mm diameter, and 60 m length. The GC program was as follows: initial oven temperature of 150 °C for 5 min, followed by ramping up to 170 °C at a rate of 3 °C/min, and held at that temperature for 5 min. The temperature was then increased up to 210 °C at a rate of 3 °C/min and held at that temperature for 5 min.

### Nile red staining

One milliliter samples were taken from MR culture and washed once with PBS, then re-suspended in 1 mL PBS. After that, 5 μL of Nile Red (0.1 mg mL^−1^ in acetone) was added into cell suspension and incubated at room temperature for 5 min in dark. Then, samples were washed twice by 100% ethanol and 75% ethyl alcohol, respectively. At last, cell suspension was imaged with a Nikon Eclipse 80i microscope (Nikon Corporation).

### PI staining

One milliliter samples were taken from MR culture (about 10^6^ individual mL^−1^) for PI staining. Then, they were centrifuged at 1000 r/min for 5 min and discarded the culture medium. Then, samples were washed twice with 3 mL PBS and centrifuged with 1 mL PBS. After centrifugation at 1000 × *g* for 5 min, PBS was discarded. Samples were re-suspended in 1 mL PBS and dyed by 3 μL PI dying at 4 °C for 20 min under dark conditions. At last, samples were imaged with a Nikon Eclipse 80i microscope (Nikon Corporation).

### Flow cytometry assays

For flow cytometry analysis, 1 mL samples were taken from MR culture and washed twice with PBS. Then, they were resuspended to an OD_600_ of 0.2 with PBS. Compensation was performed using *E. coli* that were all alive or dead when measuring *E. coli* with PI staining. For each sample, at least 20,000 counts were recorded using a 0.5 mL/s flow rate. A gate was previously designed based on forward and side scatter (>99% cells were chosen for the analysis of fluorescence density percentage). All data were exported in FCS3 format and processed using Flow Jo software (FlowJo-V10).

### Assay of field emission scanning electron microscope

Ten-milliliter samples were taken from MR culture and centrifuged at 1500 r/min for 2 min. Then, they were washed twice with PBS at pH 7.2. Finally, samples were prepared by the field emission scanning electron microscope (FEI Company, Quanta-200 and H-7650).

### Enzymatic assays

β-galactosidase gene^84^ was synthesized by GENEWIZ Biotechnology Co. Ltd. after codon optimization. As β-galactosidase can convert the *o*NPG (an analog of colorless lactose) into galactose and *o*-nitrophenol (yellow), oNPG was used as substrates for enzymatic assays and *o*-Nitrophenol can be detected at 420 nm by using a SpectraMax M3 plate reader. A 0.1 mL diluted solution of samples was added into 1.8 mL PBS (50 mM, pH 6.5) which contains 20 mM *o*NPG for reaction. The reaction solution was incubated at 50 °C for 10 min and stopped by adding 1 mL 1 M Na_2_CO_3_. All the extracellular activity represented the intracellular activity per ml medium supernatant after the cells were lysed with ultrasound.

### Assay of fluorescence intensity

The *E. coli* strains with the fluorescent protein used for assaying of fluorescence intensity were plated on the LB plates for 8 h at 37 °C. And then, a single colony was inoculated into a 50 mL fresh LB medium with 2% inoculum size (vol/vol), at 37 °C, 200 r.p.m. At last, the fluorescence of cell culture was detected by a SpectraMax M3 plate reader (Molecular Devices). The excitation and emission wavelengths of GFP were set at 480 ± 10 and 515 ± 10 nm, respectively. The excitation and emission wavelengths of mKate2 were set at 588 ± 10 and 645 ± 10 nm, respectively.

### Computational model

We described the mechanism that lead to the delay of switch time by TEVp abundance. To gain an intuitive understanding, we used a reduced model that aims to reproduce the observed switch time behavior using only the fundamental variable: the abundance of TEVp (*At*). The basic equations for strains equipped with the programmed switches were as follows (Supplementary Fig. 19b):

1 $$\frac{{dT}}{d{A}_{t}}=-0.0035\times T+0.0336$$

2 $$T=26.1868\times {e}^{-\frac{7}{2000}{A}_{t}}+9.6$$

The switch time is denoted by *T*, and the abundance of TEVp is denoted by *A*_*t*_. All simulations are carried out using Matlab.

As seen from Eqs. 1 and 2, the increase of TEVp abundance (*A*_*t*_) would cause the decline of switch time (*T*), and the rate of decline gradually slowed down. This indicated that the switch time could be delayed by reducing the abundance of TEVp, which was consistent with experimental results.

### Statistical analysis

Values are shown as mean ± s.d. from three (*n* = 3) biological replicates. Statistical significance was indicated as * for *P* < 0.05, ** for *P* < 0.01 and *** for *P* < 0.001, respectively.

### Reporting summary

Further information on research design is available in the Nature Research Reporting Summary linked to this article.

## Supplementary information

Supplementary Information

Reporting Summary

## Data Availability

Data supporting the findings of this work are available within the paper and its Supplementary Information files. A reporting summary for this Article is available as a Supplementary Information file. Source data are provided with this paper, which is also available at Figshare [https://figshare.com/articles/dataset/Source_Data_xlsx/16936924]. Source data are provided with this paper.

## Source data

Source Data
