# Supplementary material for: Reprogramming microbial populations using a programmed lysis system to improve chemical production
Source: Nat Commun. 2021 Nov 25;12:6886. doi: 10.1038/s41467-021-27226-3 (PMC8617184; doi:10.1038/s41467-021-27226-3)
Supplement: Supplementary file 1 — Supplementary Information [file 41467_2021_27226_MOESM1_ESM.pdf]

Reprogramming microbial populations using a programmed lysis system to improve chemical production

Diao *et al.*

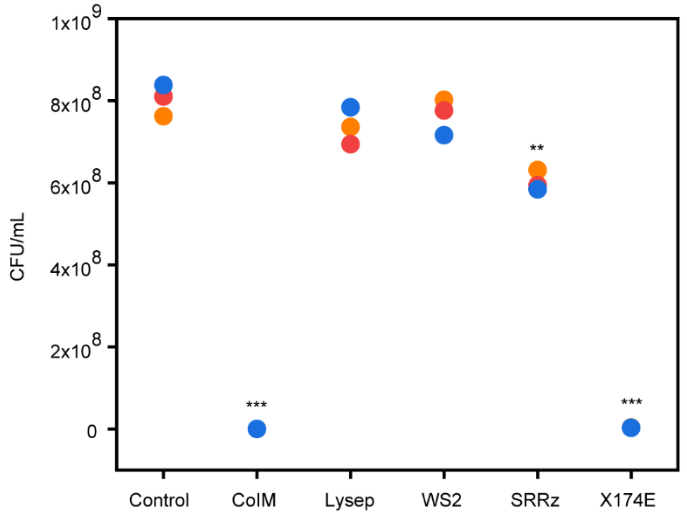

Supplementary Fig. 1. Characterization of lysis proteins. The effect of different lysis proteins on cell growth. This scatter plot illustrated the effect of different lysis proteins on CFU. (*P* values=0.000003; 0.128552; 0.316151; 0.001617; 0.000004) Strain JM109 harboring empty vector was used as the control group. The seed cultures were grown at 37 °C and 200 r.p.m. in LB medium overnight. Then they were transferred into 50 mL LB medium, and induced by 200 ng/mL aTc when OD_{600} rose to 0.6. 100 µL sample were taken and diluted by 900 µL sterile water. 100 µL of each diluted sample was spread on plate and inverted culture at 37 ℃. At last, according to the dilution factor, each the total number of colonies contained of the original samples were calculated. Two-tailed *t*-tests were used to determine statistical significance. Statistical significance was indicated as * for $P < 0.05$, ** for $P < 0.01$ and *** for $P < 0.001$, respectively. Values are shown as mean ± s.d. from three (n = 3) biological replicates. Source data are provided as a Source Data file.

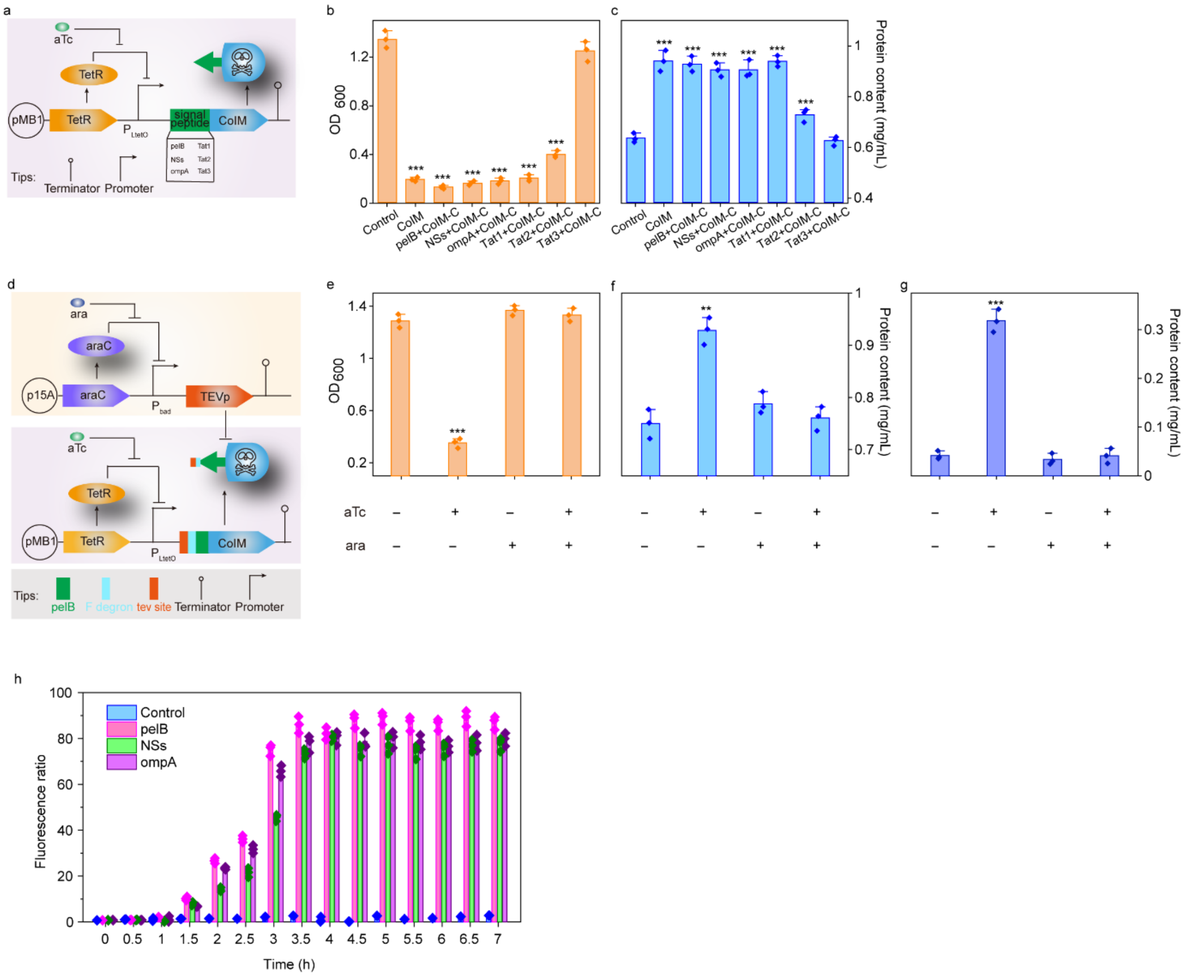

Supplementary Fig. 2. Signal peptides screening and validation of restructured ColM*. a Diagram of signal peptides screening. pelB, ompA, NSs belong to Sec-pathway and Tat1,Tat2 Tat3 belong to Tat-pathway. **b** The effect of ColM* with different signal peptides on cell growth. OD$_{600}$ showed the cell growth of *E. coli*. (*P* values=0.000010; 0.000008; 0.000009; 0.000011; 0.000012; 0.000027; 0.202409) **c** The effect of ColM* with different signal peptides on protein release in to LB medium. (*P* values=0.000306; 0.000141; 0.000144; 0.000292; 0.000050; 0.007590; 0.512552) **d** Schematic of ColM* which was controlled in a protease-trigger manner. **e** The effect of different inducers on cell growth at 2 h. (*P* values=0.000014; 0.092078; 0.333807) **f** The effect of different inducers on protein release at 2 h in LB medium. 250 mg/mL ara and 200 ng/mL aTc were added to the LB medium, respectively. (*P* values=0.001274; 0.129208; 0.628051) **g** The effect of different inducers on protein release at 2 h in M9 medium. 250 mg/mL ara and 200 ng/mL aTc were added to M9 medium, respectively. (*P* values=0.000044; 0.384444; 0.951067) **h** Fluorescence ratios were ratios of supernatant to total fluorescence, and represented the translocate capacity of different signal peptides. Strain JM109 harboring empty vector was used as the control group. Two-tailed *t*-tests were used to determine statistical significance. Values are shown as mean ± s.d. from three (n = 3) biological replicates. Statistical significance was indicated as * for $P < 0.05$, ** for $P < 0.01$ and *** for $P < 0.001$, respectively. Source data are provided as a Source Data file.

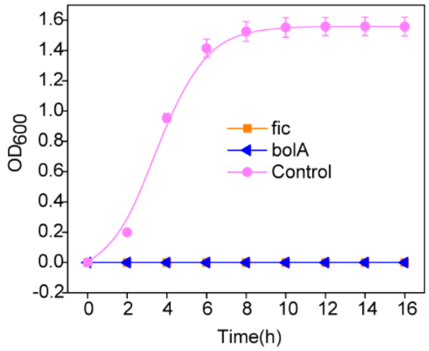

Supplementary Fig. 3. The effect of expressing ColM* with stationary phase promoters (P_{fic} and P_{bolA}) on cell growth. OD_{600} showed the growth of *E. coli*. Strains expressing ColM***** by stationary phase promoters (P_{fic} and P_{bolA}) could not grow. Strain JM109 harboring empty vector was used as the control group. The OD_{600} were measured every 2 h. All groups were grown at 37 °C and 200 r.p.m. in LB medium for 16 h. Values are shown as mean ± s.d. from three (n = 3) biological replicates. Source data are provided as a Source Data file.

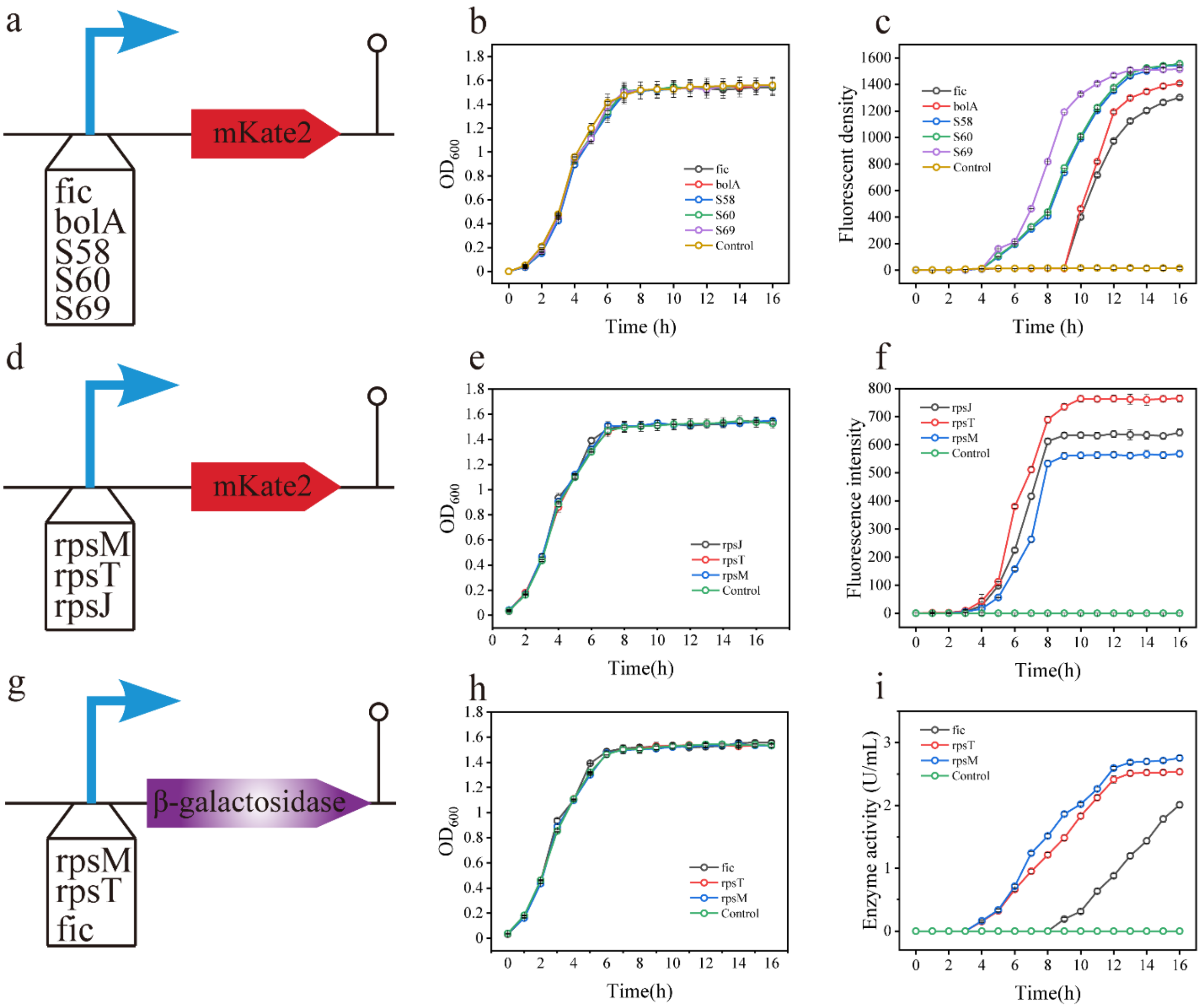

Supplementary Fig. 4. Identification of stationary phase promoters and growth phase promoters. a Diagram of identification of stationary phase promoters (P_{fic}, P_{bolA}, P_{S58}, P_{S60} and P_{S69}) by mKate2. **b** The effect of different stationary phase promoters on OD_{600}. **c** The fluorescence intensity curve represented the expression level of mKate2 by different stationary phase promoters. The increase of fluorescent density indicated that promoters turned on. **d** Diagram of identification of growth phase promoters (P_{rpsM}, P_{rpsT} and P_{rpsJ}) by mKate2. **e** The effect of different growth phase promoters on OD_{600}. **f** The fluorescence intensity curve represented the expression level of mKate2 by different growth phase promoters. The increase of fluorescent density indicated that promoters turned on. Fluorescence density was equal to fluorescence intensity divided by OD_{600}. **g** Diagram of identification of different promoters (P_{rpsM}, P_{rpsT} and P_{fic}) by β-galactosidase. **h** The effect of different promoters on OD_{600}. Strain JM109 harboring empty vector was used as the control group. **i** The fluorescence intensity curve represented the expression level of mKate2 by different growth phase promoters. The increase of enzyme activity indicated that promoters turned on. The OD_{600} and fluorescence intensity were measured every 1 h. All groups were grown at 37 °C and 200 r.p.m. in LB medium for 16 h. Values are shown as mean ± s.d. from three (n = 3) biological replicates. Source data are provided as a Source Data file.

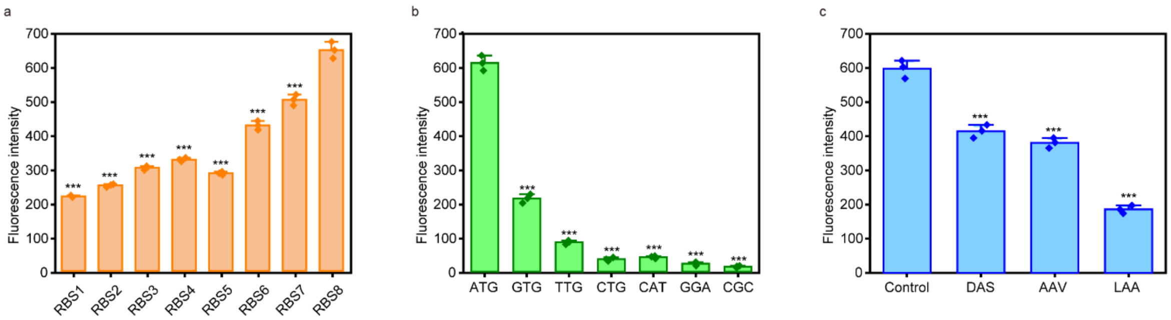

Supplementary Fig. 5. Elements of programmed switch. a The effects of different RBS on expressing fluorescent protein. Strain JM109 harboring RBS8 was the control group. (*P* values=0.000007; 0.000010; 0.000018; 0.000024; 0.000015; 0.000160; 0.000966) **b** The effects of different initial codons on expressing fluorescent protein. Strain JM109 harboring ATG was the control group. (*P* values=0.000011; 0.000002; 0.000002; 0.000002; 0.000001; 0.000001) **c** The effects of different degradation tags on expressing fluorescent protein. Strain JM109 harboring no-tags was the control group. (*P* values=0.000643; 0.000249; 0.000016) Values are shown as mean ± s.d. from three (n = 3) biological replicates. The decrease of fluorescent density indicated that abundance of fluorescent protein decreased, and the elements could decrease protein abundance. All groups were grown at 37 °C and 200 r.p.m. in LB medium for 24 h. Two-tailed *t*-tests were used to determine statistical significance. Statistical significance was indicated as * for $P < 0.05$, ** for $P < 0.01$ and *** for $P < 0.001$, respectively. Source data are provided as a Source Data file.

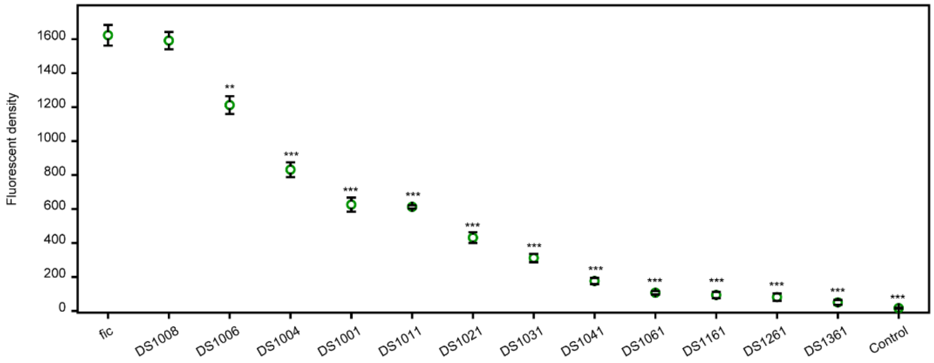

Supplementary Fig. 6. Identification of TEVp abundance. Green fluorescent protein (GFP) was fused with the C- terminal of TEVp. (*P* values=0.602704; 0.001925; 0.000116; 0.000044; 0.000020; 0.000016; 0.000009; 0.000006; 0.000004; 0.000004; 0.000005; .000003) Samples were taken at 44 h to measure OD600 and fluorescence intensity, and strain JM109 harboring empty vector was used as the control group. Fluorescence density was equal to fluorescence intensity divided by OD_{600}, and represented the abundance of GFP and TEVp. Values are shown as mean ± s.d. from three (n = 3) biological replicates. The control group was strain which did not express GFP. All groups were grown at 37 °C and 200 r.p.m. in LB medium for 44 h. Two-tailed *t*-tests were used to determine statistical significance. Statistical significance was analyzed with the fic group, and indicated as * for $P < 0.05$, ** for $P < 0.01$ and *** for $P < 0.001$, respectively. Source data are provided as a Source Data file.

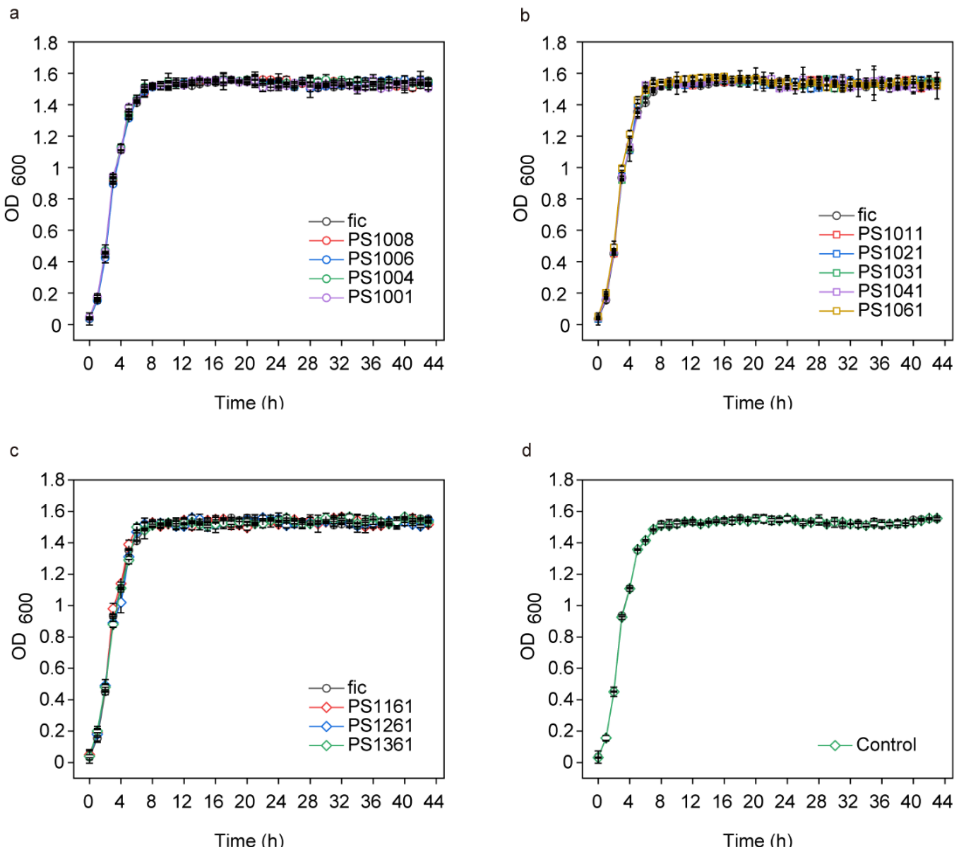

Supplementary Fig. 7. Cell growth curve of strains with different programmed switches. a Cell growth curve of strain fic, PS1008, PS1006, PS1004 and PS1001. **b** Cell growth curve of strain fic, PS1011, PS1021, PS1031, PS1041 and PS1061. **c** Cell growth curve of strain fic, PS1161, PS1261 and PS1361. **d** Cell growth curve of the control strain without proteases. All groups were grown at 37 °C and 200 r.p.m. in LB medium for 43 h. After 24 h, 0.1 mL of 100 mg/mL yeast extract was added into medium every 4 h. The OD_{600} were measured every 2 h. Values are shown as mean ± s.d. from three (n = 3) biological replicates. Source data are provided as a Source Data file.

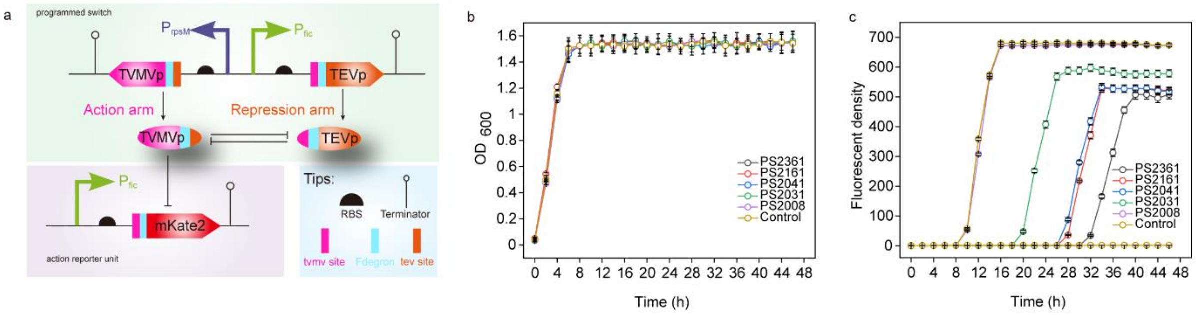

Supplementary Fig. 8. Identification the action arm of programmed switches. a Designing of the programmed switch. Characterizing programmed switch by action reporter unit. **b** Cell growth curve of strain fic, PS2161, PS2361, PS2041,PS2031 and PS2008. The strain fic was the control group, which drove TEVp by P_{fic}. **c** The fluorescence intensity curve of strain fic, PS2161, PS2361, PS2041,PS2031 and PS2008, represented the expression level of mKate2 regulated by different programmed switches. The increase of fluorescence intensity represented accumulation of mKate2, and TVMVp was cleaved by TEVp. All groups were grown at 37 °C and 200 r.p.m. in LB medium for 43 h. After 24 h, 0.1 mL of 100 mg/mL yeast extract was added into medium every 4 h. The OD_{600} and fluorescence intensity were measured every 2 h. The excitation and emission wavelengths of mKate2 were set at 588 ± 10 nm and 645 ± 10 nm, respectively. Values are shown as mean ± s.d. from three (n = 3) biological replicates. Source data are provided as a Source Data file.

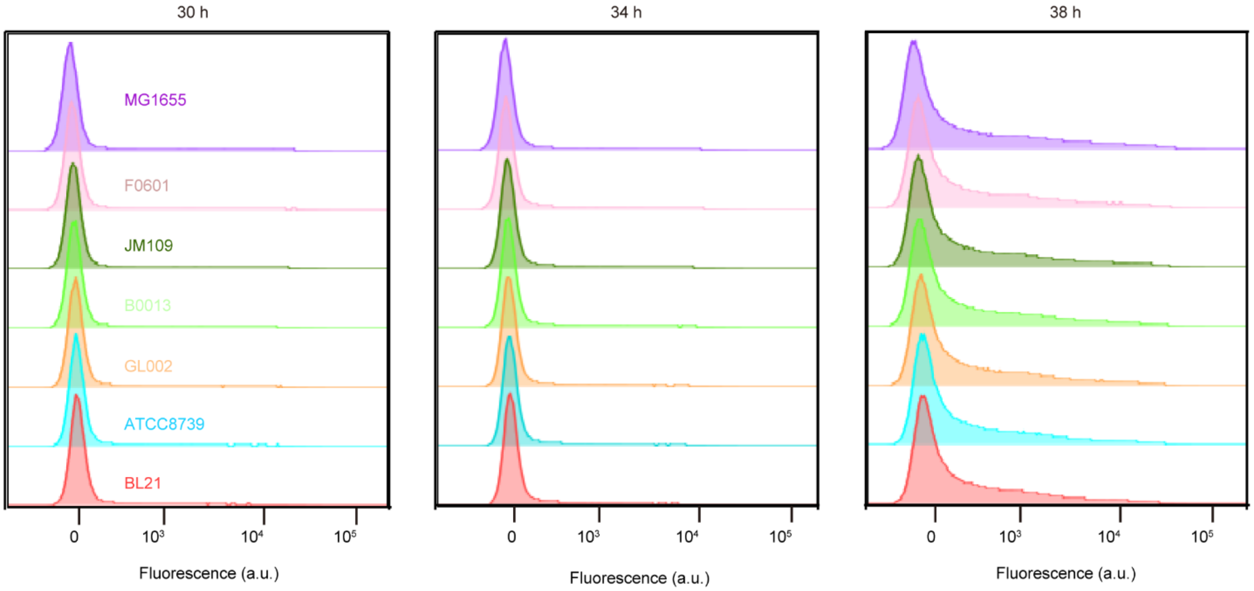

Supplementary Fig. 9. Mortality ratios comparison of different strains. Mortality ratios of different *E. coli* (without PLS) were measured by PI staining (30 h, 34 h, 38 h). The fluorescence intensity of living cells was less than 10^3, and that of dead cells was more than 10^3. All groups were grown at 37 °C and 200 r.p.m. in LB medium for 39 h. After 24 h, 0.1 mL of 100 mg/mL yeast extract was added into medium every 4 h. For each sample, at least 20,000 counts were recorded using a 0.5 mL s^{-1} flow rate. A gate was previously designed based on forward and side scatter (>99% cells were chosen for the analysis of fluorescence density percentage). All data were exported in FCS3 format and processed using Flow Jo software (FlowJo-V10).

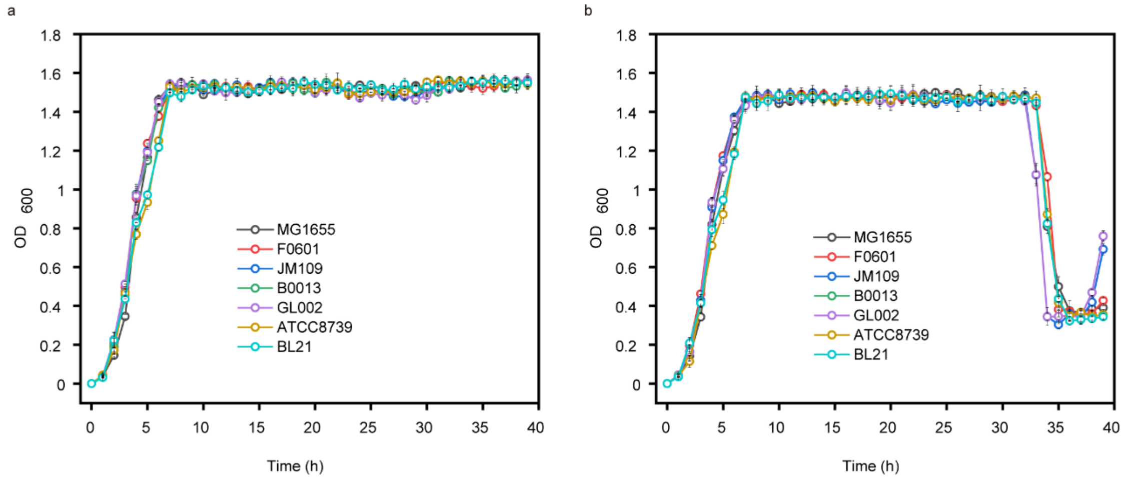

Supplementary Fig. 10. The effect of programmed lysis system (PLS) on different *E. coli*. a Cell growth curve of different *E. coli* without PLS. **b** Cell growth curve of different *E. coli* with PLS. The decrease of OD_{600} indicated cells were lysed. All groups were grown at 37 °C and 200 r.p.m. in LB medium for 39 h. After 24 h, 0.1 mL of 100 mg/mL yeast extract was added into medium every 4 h. The OD_{600} were measured every hour. Values are shown as mean ± s.d. from three (n = 3) biological replicates. Source data are provided as a Source Data file.

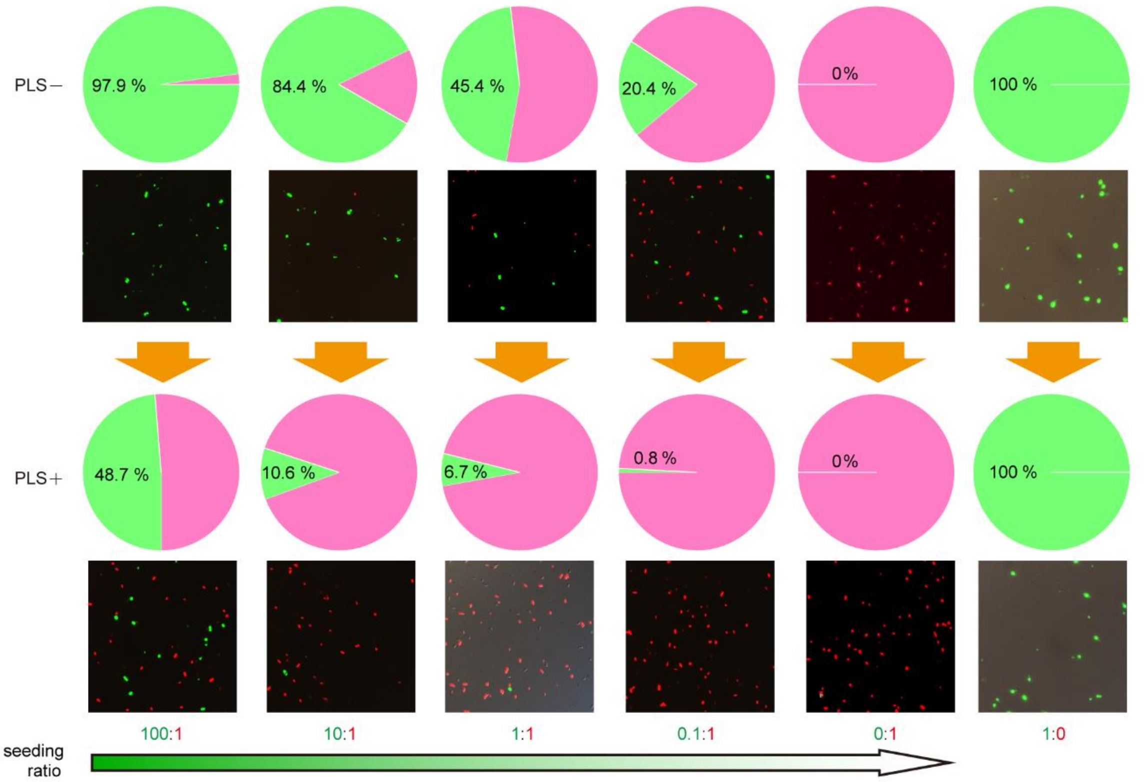

Supplementary Fig. 11. The regulation of seeding ratios and programmed lysis system on populations. The green fluorescence represented the accumulation of GFP in strain SG and SG5361. The red fluorescence represented the accumulation of mKate2 in SM. The number in the pie chart represented the proportion of *E. coli* with GFP. All cells were cultured for 60 h at 37 ℃. Their colony forming units (CFUs) were counted to calculate their ratio in the populations. Strains SG5361 and SG were seeded at different ratios (100:1, 10:1, 1:1, 0.1:1, 0:1, and 1:0) with respect to strain SM. Source data are provided as a Source Data file.

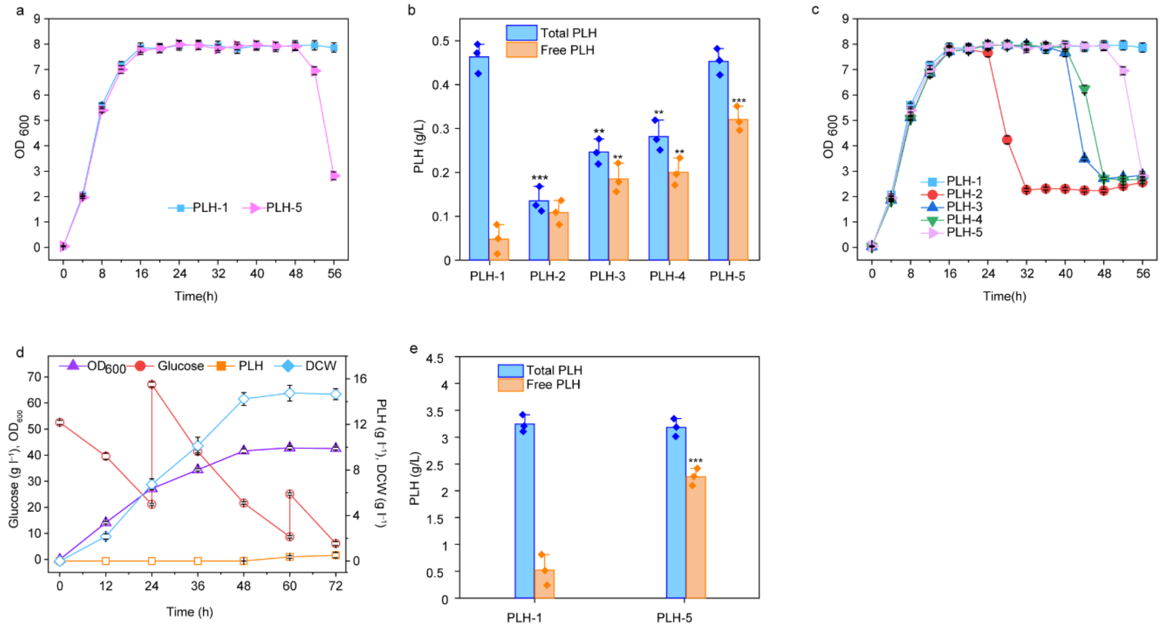

Supplementary Fig. 12. The effect of programmed lysis system on PLH production. **a** Cell growth curve of two kinds of engineered populations PLH-1 and PLH-5 in shake flasks. PLH-1 consists of B0032, and PLH-5 consists of B0033 which contains PLS based on PS3361. **b** The effect of different lysis time on PLH production. PLH-1 consists of B0032, and PLH-2 consists of B0033 which contains PLS based on PS3031; PLH-3 consists of B0033 which contains PLS based on PS3041; PLH-4 consists of B0033 which contains PLS based on PS3161; PLH-5 consists of B0033 which contains PLS based on PS3361. (*P* values=0.000230; 0.072459; 0.001107; 0.007278;0.002977; 0.004534; 0.723789; 0.000413) **c** Cell growth curve of five kinds of engineered strains with different PLS in shake flasks. **d** pH-stat fed-batch cultures of PLH-1 in a 5-L bioreactor. **e** PLH production of PLH-1 and PLH-5 in 5-L bioreactors. (*P* values=0.665258; 0.000770) Values are shown as mean ± s.d. from three (n = 3) biological replicates. Two-tailed *t*-tests were used to determine statistical significance. Statistical significance was indicated as * for 0.01 <*P* < 0.05, ** for 0.001 <*P* < 0.01 and *** for *P* < 0.001, respectively. Source data are provided as a Source Data file.

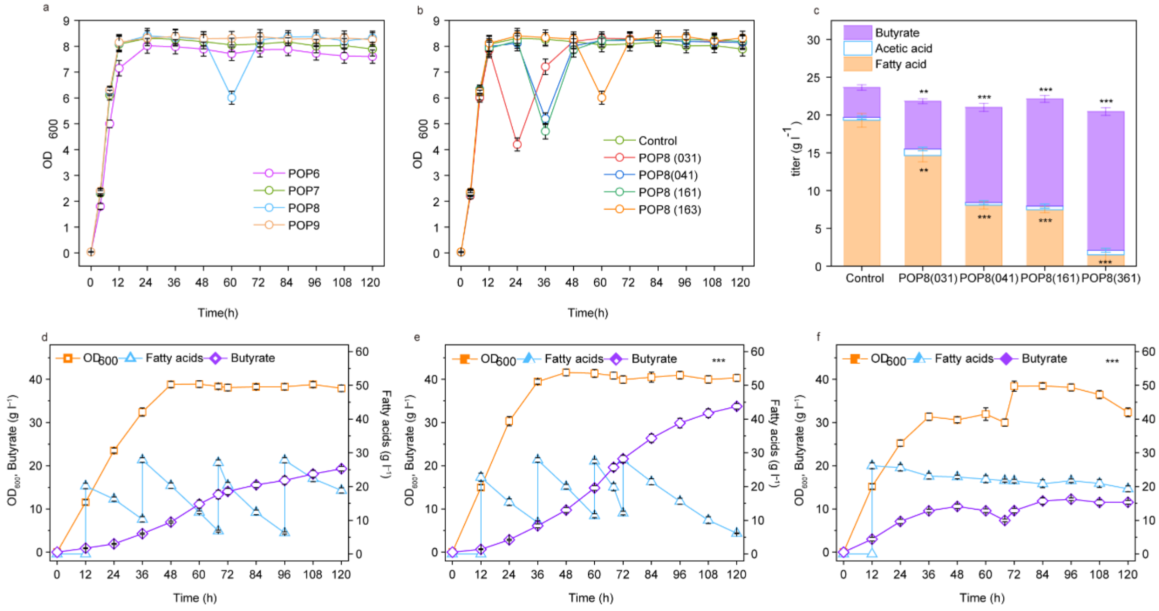

Supplementary Fig. 13. The effect of programmed lysis system on butyrate production. a Cell growth curve of four kinds of engineered populations in shake flasks. **b** Cell growth curve of four kinds of engineered populations with different PLS shake flasks. POP8 (031) was introduced PLS based on PS3031, POP8 (041) was introduced PLS based on PS3041, POP8 (161) was introduced PLS based on PS3161, POP8 (163) was introduced PLS based on PS3361, and POP7 was used as the control group. **c** The effect of different lysis time on butyrate production. (*P* values=0.001940; 0.005779; 0.000042; 0.000097; 0.000017; 0.000072; 0.000005; 0.000035) **d** pH-stat fed-batch cultures of POP6 in a 5-L bioreactor. **e** pH-stat fed-batch cultures of POP7 in a 5-L bioreactor. (*P* value=0.000016) **f** pH-stat fed-batch cultures of POP9 in a 5-L bioreactor. (*P* value=0.000078) Values are shown as mean ± s.d. from three (n = 3) biological replicates. Data are presented as mean values +/- SEM. Two-tailed *t*-tests were used to determine statistical significance. Statistical significance of butyrate was determined. Statistical significance was indicated as * for $P < 0.05$, ** for $P < 0.01$ and *** for $P < 0.001$, respectively. Source data are provided as a Source Data file.

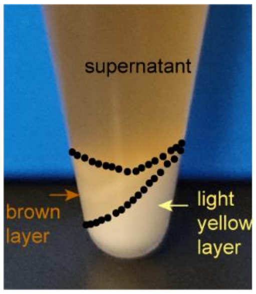

Supplementary Fig. 14. The OD_{600} determination of butyrate fermentation. The samples of culture were taken and centrifuged at 5000 r·min^{-1} for 5 min. At the bottom of tube, the sediment was divided into two layers: a brown layer (cells, upper layer) and a light yellow layer (fatty acid, lower layer). Black spots were used to make the boundaries between the supernatant, brown layer and light yellow layer more obvious. Source data are provided as a Source Data file.

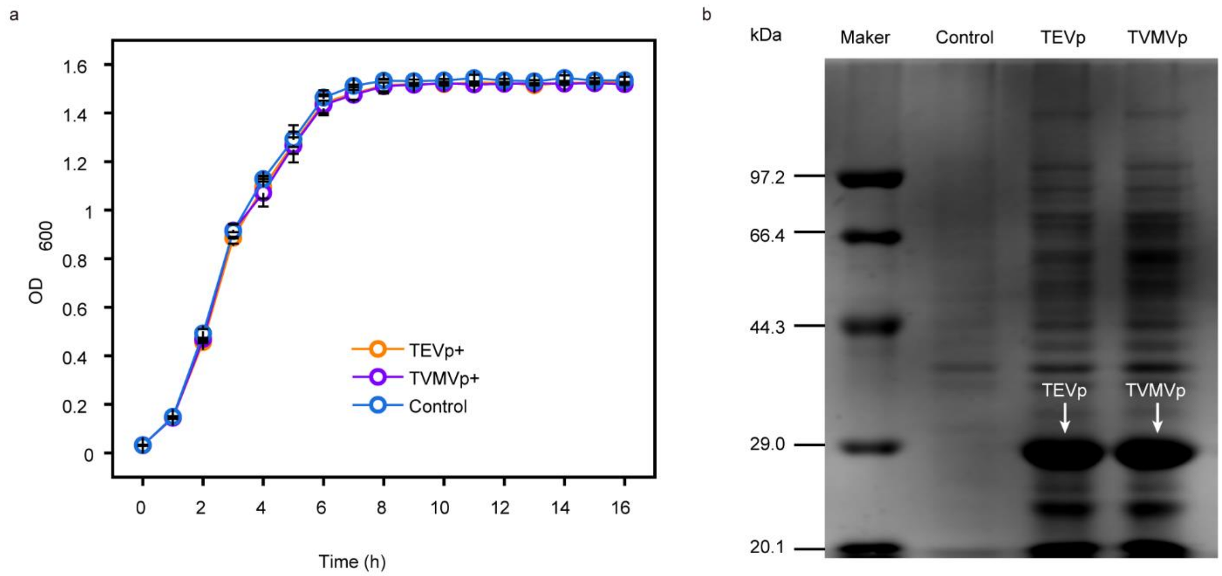

Supplementary Fig. 15. The effect of proteases TEVp and TVMVp on *E coli* growth and expression of its endogenous genes. a The effect of proteases TEVp and TVMVp on cell growth. The OD_{600} of each group were measured every 1 h. Values are shown as mean ± s.d. from three (n = 3) biological replicates. Strain JM109 harboring empty vector was used as the control group; the TEVp group was *E. coli* with TEVp expression; the TVMVp group was *E. coli* with TVMVp expression. **b** The SDS-PAGE of TEVp and TVMVp. The protein molecular weight of TEVp and TVMVp were both 29.08 kDa (white arrow). Source data are provided as a Source Data file.

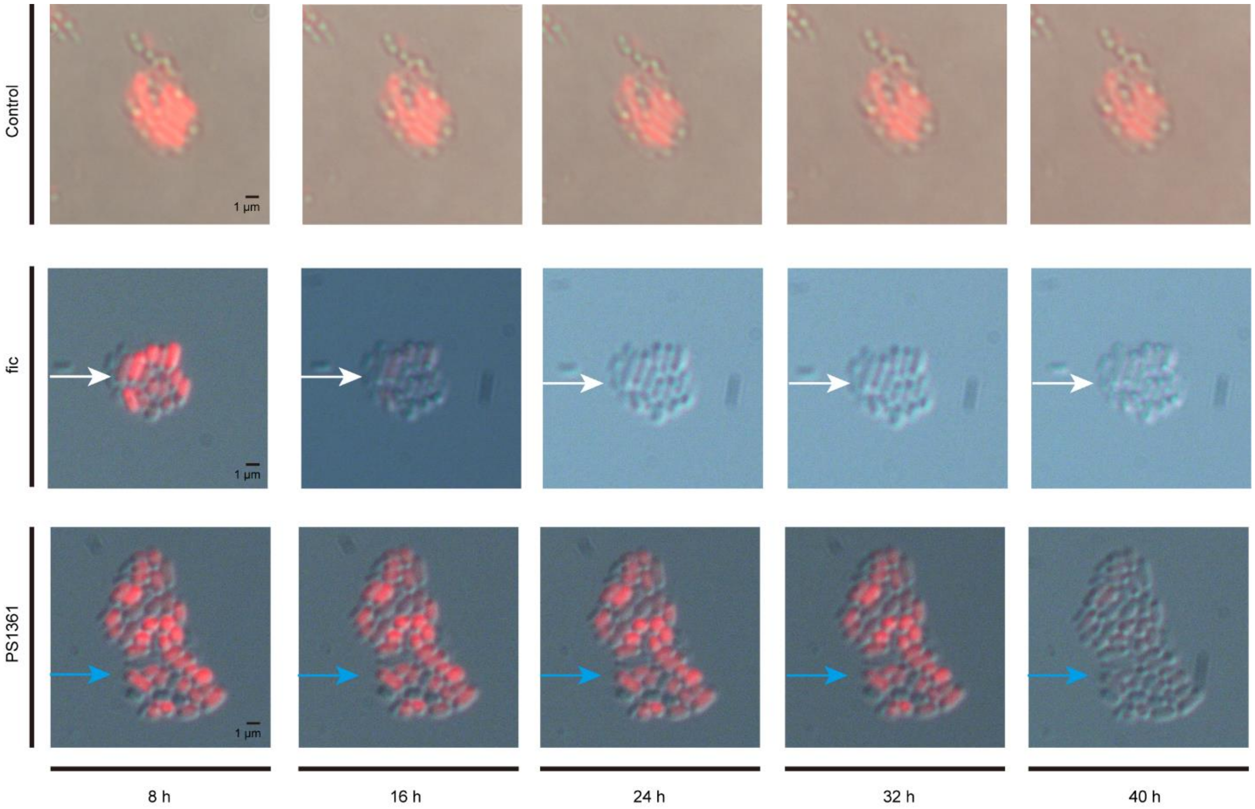

Supplementary Fig. 16. The single-cell fluorescence microscopy of the strains control, fic and PS1361. Engineered cells were immobilized on LB agar on a microscope slide, and imaged every 8 h using microscope. Strain JM109 harboring empty vector was used as the control group; the fic group was *E. coli* that drove TEVp by P_{fic} (white arrow); the PS1361 group was *E. coli* containing programmed switch (blue arrow). The fluorescence loss indicated that TEVp cleaved the repression reporter unit. Three experiments were repeated independently with similar results. Source data are provided in Source Data file.

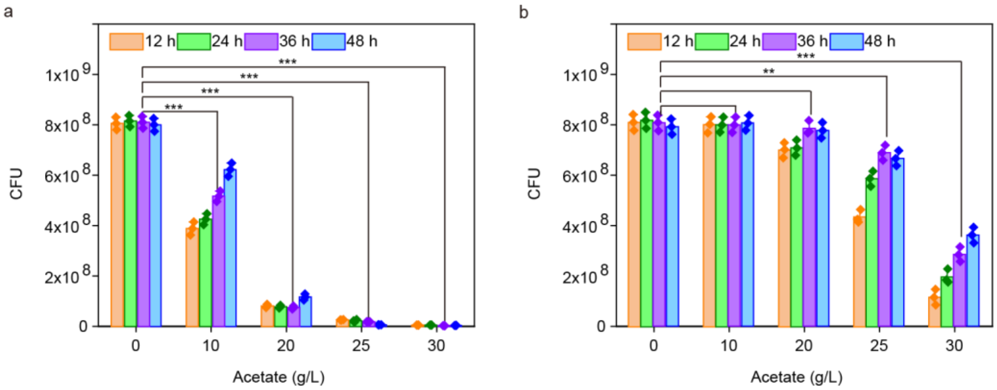

Supplementary Fig. 17. The effect of acetate concentration on the growth of *E. coli*. **a** CFU of strain F0601 under 0 g/L, 10 g/L, 20 g/L, 25 g/L, 30 g/L acetate, respectively. (*P* values=0.0000862; 0.0000008; 0.0000005; 0.0000005) **b** CFU of strain BUT004 under 0 g/L, 10 g/L, 20 g/L, 25 g/L, **3**0 g/L acetate, respectively. (*P* values=0.7678565; 0.4086567; 0.008887; 0.0000287) After inoculation, acetate with different concentration were added to LB medium, and cells were cultured at 37 °C for 12 h, 24 h, 36 h and 48 h for CFU count, respectively. Values are shown as mean ± s.d. from three (n = 3) biological replicates. Statistical significance of CFU at 36 h was determined. Two-tailed *t*-tests were used to determine statistical significance. Statistical significance is indicated as * for $P < 0.05$; ** for $P < 0.01$ and *** for $P < 0.001$, respectively. Source data are provided as a Source Data file.

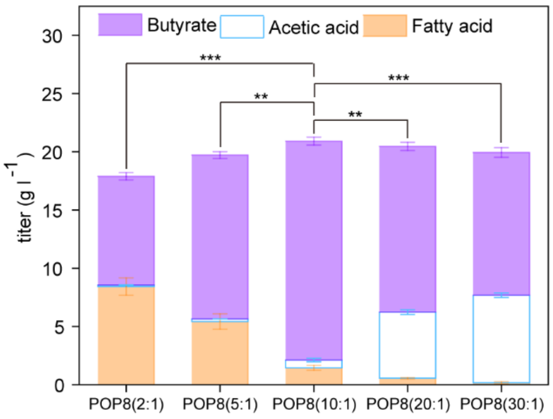

Supplementary Fig. 18. The effect of different seeding ratios on butyrate production. The seed cultures were diluted to OD_{600}=3 and mixed proportionally (strains BUT003 and BUT004 were seeded at the ratios of 2:1, 5:1, 10:1, 20:1 and 30:1) before transferred. (*P* values=0.0000082; 0.0001162; 0.0001856; 0.0000668) All the data was obtained at 37 ℃, 200 r.p.m.. Values are shown as mean ± s.d. from three (n = 3) biological replicates. Statistical significance of butyrate was determined. Two-tailed *t*-tests were used to determine statistical significance. Statistical significance is indicated as * for *P* < 0.05; ** for *P* < 0.01 and *** for *P* < 0.001, respectively. Source data are provided in Source Data file.

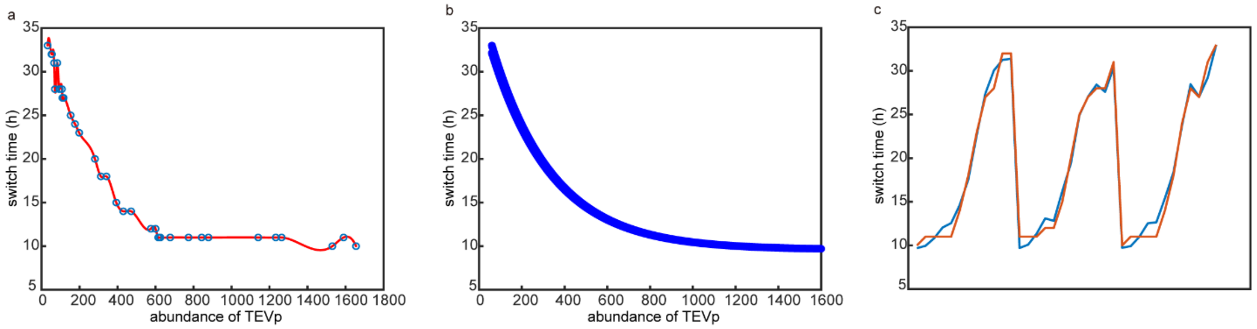

Supplementary Fig. 19. Dynamics of the switch time model. a The original behavior between the abundance of TEVp (A_t) and the switch time (T). **b** The fitting curve between the abundance of TEVp (A_t) and the switch time (T). **c** The fitting curve of the switch time (T). The red curve was original curve and the blue curve was the fitting curve. All simulations were carried out using the Matlab. To detect meaningful levels of the equation, a set of data (A_t1, $T1$) were selected and substituted A_t1 into the fitting equation to calculate $T2$. We took the relative error between the fitted value $T2$ and the true value $T1$ to determine the fit of the equation and that was $|T1\text{-}T2|/T1$. The relative error of each group of data was added and divided by the total number, and the relative error was 0.0495, which meant that the difference between the predicted value and the actual value was small, indicating that our equation had a high fitting to the original data. Source data are provided as a Source Data file.

Supplementary Table 1. Primers used in this study.

Name	Sequence(5'-3')	Purpose
tesB-F	atgagtcaggcgctaaaaaatttactg	*tesB* cloning in pTrcHisA
tesB-R	aaggggtgatgcgtaatcacaattaa	*tesB* cloning in pTrcHisA
atoB-F	atgaaaaattgtgtcatcgtcagtgc	*atoB* cloning in pTrcHisA
atoB-R	gcgatggtgattgaacggttgaattaa	*atoB* cloning in pTrcHisA
ter-F	atgatagtaaaagcaaagtttgtaaaaggattt	*ter(Ca)* cloning in pCloDF13
ter-R	cctggatatagaattattaagaaaattagaacctt aa	*ter(Ca)* cloning in pCloDF13
hbd-F	atgaaaaaggtatgtgttataggtgcag	*hbd(Ca)* cloning in pCloDF13
hbd-R	ggtttctacgattatttcaaaataa	*hbd(Ca)* cloning in pCloDF13
YZ-tet-F	ccttcgattccgacctcat	DNA fragment inserting confirmation
YZ-tet-R	gttcaccgacaaacaacagata	DNA fragment inserting confirmation
Tev-F	ttcttattcgtgcaagaactgggtgaaagcctgttt aaaggt	Tev cloning in pTet-1
Tev-R	ctgcagatgaatttcttcgctacccggatcctggc tgtggt	Tev cloning in pTet-1
Tvmv-F	cgggatcctggtagcgaaaacctgtattttcagtt cttattcgtgcaagaactgagta	Tvmv cloning in pTet-1
Tvmv-R	cccaagcttttattcaaccagggtaaaactgc	Tvmv cloning in pTet-1
ColM-C-F	atgagcggtaatgtcactacacc	ColM-C cloning in pTet-1
ColM-C-R	aaattcacattaaagaaagtggtaagcgataa	ColM-C cloning in pTet-1
QC-fadR-F	ttgaagcaaaaacggccaaagt	SgRNA construction for *fadR* deletion
QC-fadR-R	attactggtatccggaattcacaataagtaa	SgRNA construction for *fadR* deletion
QC-PTA-F	gtgtcccgtattattatgctgatccct	SgRNA construction for *PTA* deletion
QC-PTA-R	ttcagtctgcacagcagcagtaa	SgRNA construction for *PTA* deletion
sgRNA-R	actagtattatacctaggactgagc	SgRNA construction

Supplementary Table2. Signal peptides used in this study.

Signal peptide	Sequence
Tat1	atgtctctgtctcgtcgtcagttcatccaggcttctggtatcgctctgtgcgctggtgctgttccgctgaaagcttctgct
Tat2	atgcagacccgtcgtgttgttctgaaatctgctgctgctgctggtaccctgctgggtggtctggctggttgcgcttctgttgctggttct
Tat3	atgggtaccgaagtttctcgtcgtaaactgatgaaaggtgctgctgtttctggtggtgctctggctctgccggctctgggtgctccgccggctaccgctgctccggctgctggtccggaagacctgccgggtccggctgctgct
ompA	atgaaaaagacagctatcgcgattgcagtggcactggcaggtttcgctaccgtcgctcaggctggatccgca
Nss	atgatgattactctgcgcaaacttcctctggcggttgccgtcgcagcgggcgtaatgtctgctcaggct
pelB	atgaaatacctgctgccgaccgctgctgctggtctgctgctcctcgctgcccagccggcgatggcc

Supplementary Table 3. Plasmids used in the programmed switch.

Signal peptide	Sequence
pTet-1	P_{tet}, p15A ori, Cm^R, tetR
pTet-2	P_{rpsM} and P_{fic}, p15A ori, Cm^R
pTrcHisA-1	P_{Trc}, pBR322 ori, Amp^R, $LacI^q$
pTrcHisA-2	P_{rpsT}, pBR322 ori, Amp^R
pTrcHisA-3	P_{fic}, pBR322 ori, Amp^R
pTet-*tev*	TEVp expression with RBS8 on pTet-1
pTet-*tvmv*	TVMVp expression with RBS8 on pTet-1
pTet-008*tev-tvmv*	TEVp expression with RBS8, ATG; TVMVp expression with RBS8 on pTet-2
pTet-006*tev-tvmv*	TEVp expression with RBS6, ATG; TVMVp expression with RBS8 on pTet-2
pTet-004*tev-tvmv*	TEVp expression with RBS4, ATG; TVMVp expression with RBS8 on pTet-2
pTet-001*tev-tvmv*	TEVp expression with RBS1, ATG; TVMVp expression with RBS8 on pTet-2
pTet-011*tev-tvmv*	TEVp expression with RBS1, ATG; TVMVp expression with RBS8 on pTet-2
pTet-021*tev-tvmv*	TEVp expression with RBS1, GTG; TVMVp expression with RBS8 on pTet-2
pTet-031*tev-tvmv*	TEVp expression with RBS1, TTG; TVMVp expression with RBS8 on pTet-2
pTet-041*tev-tvmv*	TEVp expression with RBS1, CTG; TVMVp expression with RBS8 on pTet-2
pTet-061*tev-tvmv*	TEVp expression with RBS1, GGA; TVMVp expression with RBS8 on pTet-2
pTet-161*tev-tvmv*	TEVp expression with RBS1, GGA and fused with DAS; TVMVp expression with RBS8 on pTet-2
pTet-261*tev-tvmv*	TEVp expression with RBS1, GGA and fused with AAV; TVMVp expression with RBS8 on pTet-2
pTet-361*tev-tvmv*	TEVp expression with RBS1, GGA and fused with LAA; TVMVp expression with RBS8 on pTet-2
pPLS	ColM expression with RBS1 on pTet-361*tev-tvmv*
pTrcHisA-(teF)mKate2	Fused protein containing tev site, F degron, mKate2 was inserted in pTrcHisA-2
pTrcHisA-(tvF)mKate2	Fused protein containing tvmv site, F degron, mKate2 was inserted in pTrcHisA-3
pTrcHisA-(tvF)gal	Fused protein containing tvmv site, F degron, β-galactosidase was inserted in pTrcHisA-3

Supplementary Table 4. Lysis genes used in this study.

Gene	Sequence
ColM (C-terminal)	tcacacaggaaagaagcttatgaaatacctgctgccgaccgctgctgctggtctgctgctcctcgctgcccagccggcgatggccctgcagatgagcggtaatgtcactacaccaattgtggcgcttgctcactatttatggggtaatggcgctgaaaggagcgttaatatcgccaacattggtcttaaaatttcccctatgaaaattaatcagataaaagacattataaaatctggtgtagtaggtacattccctgtttctacaaagttcacacatgccactggtgattataatgttattaccggtgcatatcttggtaatatcacactgaaaacagaaggtactttaactatctctgccaatggctcctggacttacaatggcgttgttcgttcatatgatgataaatacgattttaacgccagcactcaccgtggcattatcggagagtcgctcacaaggctcggggcgatgttttctggtaaagagtaccagatactgcttcctggtgaaattcacattaaagaaagtggtaagcgataa
Lysep	atgtcacacaggaaagaagcttatgaaatacctgctgccgaccgctgctgctggtctgctgctcctcgctgcccagccggcgatggccacgatcggctagaatcggcgctagatcgatatagctctgactcgatcgctagaatatagcgagagctctcgctcgatatcgctagctagagatctctcgctagctcgctcgctcgctatagctagctagcgcgctcgctcgctagctcatcgctcgctcgatatagatagatacgatagacgatcgcgccctcgctagattagatcgctcgatcgctagcgcgctcgctcgctcgctatagatagatcgctcgctagatcgctctcgctagctcgcgctagctatatagctcgctcgctcgatcgtagagatatacagacagatagatctcgcgattatatatagagggacgctcttcccgcttagatcgctagctcgctcgctcgatagctagctcgctcgctcgatagatacatacatcatagctcgctcgctagacatacatacagatacagatacagatagacagatctcgctcgctgcctgtgtatatagatgacagatgacgatgacgatacgctcgctagacgatcgcagatcgatacagatacagatacaggagatcgctagctcgatcgtaa
X174E	atgcagacccgtcgtgttgttctgaaatctgctgctgctgctggtaccctgctgggtggtctggctggttgcgcttctgttgctggttctctgcagatgagcggtaatgtcactacaccaattgtggcgcttgctcactatttatggggtaatggcgctgaaaggagcgttaatatcgccaacattggtcttaaaatttcccctatgaaaattaatcagataaaagacattataaaatctggtgtagtaggtacattccctgtttctacaaagttcacacatgccactggtgattataatgttattaccggtgcatatcttggtaatatcacactgaaaacagaaggtactttaactatctctgccaatggctcctggacttacaatggcgttgttcgttcatatgatgataaatacgattttaacgccagcactcaccgtggcattatcggagagtcgctcacaaggctcggggcgatgttttctggtaaagagtaccagatactgcttcctggtgaaattcacattaaagaaagtggtaagcgataagcggccgcataa
SRRz	atgaagatgccagaaaaacatgacctgttggccgccattctcgcggcaaaggaacaaggcatcggggcaatccttgcgtttgcaatggcgtaccttcgcggcagatataatggcggtgcgtttacaaaaacagtaatcgacgcaacgatgtgcgccattatcgcctggttcattcgtgaccttctcgacttcgccggactaagtagcaatctcgcttatataacgagcgtgtttatcggctacatcggtactgactcgattggttcgcttatcaaacgcttcgctgctaaaaaagccggagtagaagatggtagaaatcaataatcaacgtaaggcgttcctcgatatgctggcgtggtcggagggaactgataacggacgtcagaaaaccagaaatcatggttatgacgtcattgtaggcggagagctatttactgattactccgatcaccctcgcaaacttgtcacgctaaacccaaaactcaaatcaacaggcgccggacgctaccagcttctttcccgttggtgggatgcctaccgcaagcagcttggcctgaaagacttctctccgaaaagtcaggacgctgtggcattgcagcagattaaggagcgtggcgctttacctatgattgatcgtggtgatatccgtcaggcaatcgaccgttgcagcaatatctgggcttcactgccgggcgctggttatggtcagttcgagcataaggctgacagcctgattgcaaaattcaaagaagcgggcggaacggtcagagagattgatgtatgagcagagtcaccgcgattatctccgctctggttatctgcatcatcgtctgcctgtcatgggctgttaatcattaccgtgataacgccattacctacaaagcccagcgcgacaaaaatgccagagaactgaagctggcgaacgcggcaattactgacatgcagatgcgtcagcgtgatgttgctgcgctcgatgcaaaatacacgaaggagttagctgatgctaaagctgaaaatgatgctctgcgtgatgatgttgccgctggtcgtcgtcggttgcacatcaaagcagtctgtcagtcagtgcgtgaagccaccaccgcctccggcgtggataatgcagcctcccccgactggcagacaccgctgaacgggattatttcaccctcagagagaggctgatcactatgcaaaaacaactggaaggaacccagaagtatattaatgagcagtgcagatag
MS2	Atggccagcaatttcacccagttcgttctggttgataatggcggcaccggcgatgttaccgttgccccgagcaatttcgccaatggcgttgccgaatggatcagcagcaatagccgcagccaggcctacaaagttacctgcagcgttcgccagagcagcgcccagaatcgcaaatacaccatcaaagttgaagttccgaaagttgccacccagaccgttggcggcgttgaactgccggttgccgcctggcgcagctacctgaatatggaactgaccatcccgatcttcgccaccaatagcgattgcgaactgatcgttaaagccatgcagggcctgctgaaagatggcaatccgatcccgagcgccatcgccgccaatagcggcatctactaa

Supplementary Table 5. Degron sequences used in this study.

Degron name	Sequence
LAA	AANDENYALAA
DAS	AANDENYADAS
DAS	AANDENYNYADAS

Supplementary Table 6. RBS sequences used in this study.

RBS name	Sequence
RBS1	agggacaggat
RBS2	tcacacaggac
RBS3	gaaagaggggacaa
RBS4	gaaagagagtggaact
RBS5	gaaagaagagactc
RBS6	tcacacaggaaag
RBS7	tagcaggaggaa
RBS8	aaagaggagaaa

Supplementary Table 7. Plasmids used for PLH and butyrate production.

Plasmid name	Plasmid characteristics
pTCH	Gene *ter*, *crt* and *hbd* were co-expressed on pJ01
pTCH(zu)	pTCH, *ter*, *crt* and *hbd* expression with P_{J23105} promoter, RBS4 on pJ01
pATTCH(zu)	pTCH(zu), *atoB* and *tesB* co-expression with P_{J23101} promoter, RBS6 on pJ01
pAATTCH(zu)	pATTCH(zu), *ACS* expression with P_{J23119} promoter, RBS8 on pJ01
pT-DEBA	Gene *FadD*, *FadE*, *FadB* and *FadA* was inserted in pTrcHisA
pT-PADEBA	pT-DEBA, *PTA* and *ACK* co-expression with P_{J23108} promoter, RBS6 on pTrcHisA
pABCP	Gene *phaA*, *phaB*, *phaC* and *pct* was inserted in pTrcHisA
pPLS	*ColM* expression with P_{fic} promoter on p361
pGFP	GFP expression with P_{J23101} promoter, RBS6 on pJ01
pMk	mKate2 expression with P_{J23101} promoter, RBS6 on pJ01

Supplementary Table 8. Genotypes of strains constructed in this paper.

	Strains	Relevant characteristics	Reference
1	*E.coli* JM109	Wild type *E.coli* JM109	ATCC
2	*E.coli* 8739	Wild type *E.coli* 8739	ATCC
3	GL0002	*E.coli* 8739 △*ackA,* △*adhE*	[1]
4	F0601	*E.coli* W3110 △*ldhA,* △*pflB,* △*poxB,* △*adhE,* △*pta-ackA*	[2]
5	PS1008	*E.coli* JM109 harboring pTet-008*tev-tvmv*, pTrcHisA-(teF)mKate2	This study
6	PS1006	*E.coli* JM109 harboring pTet-006*tev-tvmv*, pTrcHisA-(teF)mKate2	This study
7	PS1004	*E.coli* JM109 harboring pTet-004*tev-tvmv*, pTrcHisA-(teF)mKate2	This study
8	PS1001	*E.coli* JM109 harboring pTet-001*tev-tvmv*, pTrcHisA-(teF)mKate2	This study
9	PS1011	*E.coli* JM109 harboring pTet-011*tev-tvmv*, pTrcHisA-(teF)mKate2	This study
10	PS1021	*E.coli* JM109 harboring pTet-021*tev-tvmv*, pTrcHisA-(teF)mKate2	This study
11	PS1031	*E.coli* JM109 harboring pTet-031*tev-tvmv*, pTrcHisA-(teF)mKate2	This study
12	PS1041	*E.coli* JM109 harboring pTet-041*tev-tvmv*, pTrcHisA-(teF)mKate2	This study
13	PS1061	*E.coli* JM109 harboring pTet-061*tev-tvmv*, pTrcHisA-(teF)mKate2	This study
14	PS1161	*E.coli* JM109 harboring pTet-161*tev-tvmv*, pTrcHisA-(teF)mKate2	This study
15	PS1261	*E.coli* JM109 harboring pTet-261*tev-tvmv*, pTrcHisA-(teF)mKate2	This study
16	PS1361	*E.coli* JM109 harboring pTet-361*tev-tvmv*, pTrcHisA-(teF)mKate2	This study
17	PS2031	*E.coli* JM109 harboring pTet-031*tev-tvmv*, pTrcHisA-(tvF)mKate2	This study
18	PS2041	*E.coli* JM109 harboring pTet-041*tev-tvmv*, pTrcHisA-(tvF)mKate2	This study

19	PS2161	*E.coli* JM109 harboring pTet-161*tev-tvmv*, pTrcHisA-(tvF)mKate2	This study
20	PS3008	*E.coli* JM109 harboring pTet-008*tev-tvmv*, pTrcHisA-(tvF)gal	This study
21	PS3031	*E.coli* JM109 harboring pTet-031*tev-tvmv*, pTrcHisA-(tvF)gal	This study
22	PS3041	*E.coli* JM109 harboring pTet-041*tev-tvmv*, pTrcHisA-(tvF)gal	This study
23	PS3161	*E.coli* JM109 harboring pTet-161*tev-tvmv*, pTrcHisA-(tvF)gal	This study
24	PS3361	*E.coli* JM109 harboring pTet-361*tev-tvmv*, pTrcHisA-(tvF)gal	This study
25	SG5361	JM109 harboring pPLS, pGFP	This study
26	SG	JM109 harboring pGFP	This study
27	SM	JM109 harboring pMk	This study
28	B0032	*E.coli* 8739 △*ackA,* △*adhE*, harboring pABCP	[3]
29	B0033	B0032 harboring pPLS	This study
30	F0602	F0601 *pta*:: *acs*, *fadE*::*tesA*	This study
31	BUT001	F0602 harboring pT-DEBA, pATTCH(zu), pTCH	This study
32	BUT002	8739 harboring pT-DEBA	This study
33	BUT003	8739 harboring pT-DEBA, pPLS	This study
34	BUT004	F0602 harboring pATTCH(zu), pTCH	This study

Supplementary Table 9. **PCR primers used to DNA manipulation.**

	Primer	Sequence
1	*atoB*-1	ATGAAAAATTGTGTCATCGTCAGTGC
2	*atoB*-2	TTAATTCAACCGTTCAATCACCATCGC
3	*tesB*-1	ATGAGTCAGGCGCTAAAAAATTTACTGAC
4	*tesB*-2	TTAATTGTGATTACGCATCACCCCTTC
5	*ter*-1	ATGATAGTAAAAGCAAAGTTTGTAAAAGGATTTATCA
6	*ter*-2	TTAAGGTTCTAATTTTCTTAATAATTCTATATCC
7	*hbd*-1	ATGAAAAAGGTATGTGTTATAGGTGCA
8	*hbd*-2	TTATTTTGAATAATCGTAGAAACCTTTTC
9	*Crt*-1	ATGGAACTAAACAATGTCATCCTTGAA
10	*Crt*-2	CTATCTATTTTTGAAGCCTTCAATTTT
11	*phaA*-1	ATGACTGACGTTGTCATCGTATCC
12	*phaA*-2	TTATTTGCGCTCGACTGCCAGC
13	*phaB*-1	ATGACTCAGCGCATTGCGTA
14	*phaB*-2	TCAGCCCATATGCAGGCCGCCGT
15	*fadD*-1	ATGAAGAAGGTTTGGCTTAACCGTTATC
16	*fadD*-2	TCAGGCTTTATTGTCCACTTTGCCG
17	*fadE*-1	ATGATGATTTTGAGTATTCTCGCTACG
18	*fadE*-2	TTACGCGGCTTCAACTTTCCG
19	*fadB*-1	ATGATTTACGAAGGTAAAGCCATCACG
20	*fadB*-2	TTAACCGAAGAAGCTCTGGCCG
21	*tesA*-1	ATGATGAACTTCAACAATGTTTTCCGCT
22	*tesA*-2	TTATGAGTCATGATTTACTAAAGGCTGCAA

Supplementary references

1. Guo L, *et al.* Enhancement of malate production through engineering of the periplasmic rTCA pathway in *Escherichia coli*. *Biotechnol. Bioeng.* **115**, 1571-1580 (2018).

2. Dong X, *et al.* Metabolic engineering of *Escherichia coli* W3110 to produce L-malate. *Biotechnol. Bioeng.* **114**, 656-664 (2017).

3. Guo L, *et al.* Engineering *Escherichia coli* lifespan for enhancing chemical production. *Nat. Catal.* **3**, 307-318 (2020).
